# Comparison between High-Power Short-Duration and Conventional Ablation Strategy in Atrial Fibrillation: An Updated Meta-Analysis

**DOI:** 10.1155/2022/1065077

**Published:** 2022-07-29

**Authors:** Mohan Li, Yingxu Ma, Qiuzhen Lin, Yunying Huang, Yaozhong Liu, Tao Tu, Qiming Liu

**Affiliations:** ^1^Department of Geriatrics, Peking Union Medical College Hospital, Chinese Academy of Medical Sciences, Beijing 100730, China; ^2^Department of Cardiovascular Medicine, Second Xiangya Hospital, Central South University, Changsha 410011, China

## Abstract

High-power short-duration (HPSD) setting during radiofrequency ablation has become an attempt to improve atrial fibrillation (AF) treatment outcomes. This study ought to compare the efficacy, safety, and effectiveness between HPSD and conventional settings. PubMed, Embase, and Cochrane Library were searched. Studies that compared HPSD and conventional radiofrequency ablation settings in AF patients were included while studies performed additional ablations on nonpulmonary vein targets without clear recording were excluded. Data were pooled with random-effect model. Efficacy endpoints include first-pass pulmonary vein isolation (PVI), acute pulmonary vein (PV) reconnection, free from AF, and free from atrial tachycardia (AT) during follow-up. Safety endpoints include esophagus injury rate and major complication rate. Effectiveness endpoints include complete PVI rate, total procedure time, PVI time, and PVI radiofrequency ablation (PVI RF) time. We included 22 studies with 3867 atrial fibrillation patients in total (2393 patients received HPSD radiofrequency ablation). Perioperatively, the HPSD group showed a higher first-pass PVI rate (risk ratio, RR = 1.10, *P* = 0.0001) and less acute PV reconnection rate (RR = 0.56, *P* = 0.0004) than the conventional group. During follow-up, free from AF (RR = 1.11, *P* = 0.16) or AT (RR = 1.06, *P* = 0.24) rate did not differ between HPSD and conventional groups 6-month postsurgery. However, the HPSD group showed both higher free from AF (RR = 1.17, *P* = 0.0003) and AT (RR = 1.11, *P* < 0.0001) rate than the conventional group 12-month postsurgery. The esophagus injury (RR = 0.99, *P* = 0.98) and major complications (RR = 0.76, *P* = 0.70) rates did not differ between the two groups. The HPSD group took shorter total procedure time (MD = −33.71 95% CI: -43.10 to -24.33, *P* < 0.00001), PVI time (MD = −21.60 95% CI: -25.00 to -18.21, *P* < 0.00001), and PVI RF time (MD = −13.72, 95% CI: -14.45 to -13.00, *P* < 0.00001) than conventional groups while complete procedure rate did not differ between two groups (RR = 1.00, *P* = 0.93). HPSD setting during AF radiofrequency ablation has better effectiveness, efficacy, and similar safety compared with the conventional setting.

## 1. Introduction

Radiofrequency catheter ablation has been proven to be an effective treatment for atrial fibrillation (AF). Pulmonary vein isolation (PVI), aiming at blocking pulmonary veins (PVs) generated ectopic current conduction into the left atrial, is the most important radiofrequency ablation (RFA) technique for treating AF [1, 2]. PV reconnection was recognized as the major cause of AF recurrence after PVI and the failure of AF catheter ablation, which makes durable and transmural lesions formed during ablation critical to long-term successful PVI. Multiple factors were correlated with high-quality RFA lesions, including power, contact force (CF), radiofrequency ablation time to form each lesion, impedance drop, and ablation site temperature. Novel indices were brought out by coordinating the abovementioned factors, like ablation index (AI) or lesion size index (LSI), aiming at achieving better AF radiofrequency ablation outcomes [3–6].

Power, as one of the most easily manipulatable parameters during the procedure, drew the interest of researchers for a while. The first attempt of increasing RFA power to 45 W and shortening ablation time was launched in 2006 by Nilsson and his colleagues [7]. Since then, high-power short-duration (HPSD) as a novel RFA strategy has been broadly studied in both animal models and clinical cases. By increasing resistive heating in the lesion core and reducing the lesion area caused by conductive heating, HPSD strategy creates shallower but wider lesions in both in silico and animal models compared with the conventional technique or so-called lower-power longer-duration (LPLD) strategy [8, 9]. Several meta-analyses about the efficacy of HPSD strategy have been conducted but provided different results. Higher free from or less recurrence of atrial tachycardia (AT)/atrial flutter in HPSD group has been reported in some literature, while others disagreed [10–13]. Additional ablations than PVI during RFA procedure were performed at operators' discretion for different patients, which usually included left atrial posterior wall box isolation, linear isolation, and tricuspid isthmus ablation. These additional ablations on non-PV targets could be one of the sources of heterogeneity but have been ignored by most existing researches. For this reason, we conducted this updated meta-analysis comparing the efficacy, safety, and effectiveness between the HPSD and conventional strategies. We added the most updated studies with more precise sorting approaches, like distinguishing PVI from additional ablations and pooling AF or AT recurrence data according to whether additional ablations were performed or not. Different subgroup analyses were also performed in this study to further explore the effect of power, integral parameters, and antiarrhythmic drugs on AF rhythm management.

## 2. Methods

### 2.1. Studies Selection

Search has been conducted in PubMed, Embase, and Cochrane Library from inception to January 2022 with language limited to English. The keywords for searching included “atrial fibrillation,” “radiofrequency ablation,” “catheter ablation,” and “power” with their MeSH terms or Emtree checked corresponding to different databases. Detailed searching strategies are available in Supplementary Table S1-S3. Further literature retrieval was performed by screening the reference list of included articles. Two authors independently performed searches, screened titles, abstracts, and full texts when needed. Disagreements were solved through consensus.

The inclusion criteria for this study are as follows: (1) patients have been diagnosed with nonvalvular AF and underwent RFA for the first time; (2) PVI was performed by catheter radiofrequency ablation; (3) randomized controlled trials, nonrandomized trials, and observational studies included both HPSD and conventional (or low-power long-duration) treatment groups; (4) studies with at least 3-month follow-up after RFA. The exclusion criteria for this study are as follows: (1) additional RFA than PVI was performed without detailed reports for each independent ablation procedure; (2) studies with equivocal design or results reported; (3) single-armed studies; and (4) animal studies, case reports, conference abstracts, or literature lacking endpoints of interest.

The endpoints of interest in this study are efficacy endpoints, safety endpoints, and effectiveness endpoints. Efficacy endpoints included first-pass PVI, acute PV reconnection, free from AF or AT 6, or 12 months postsurgery. Safety endpoints included esophageal injury and procedure-related major complications. More specifically, we defined major complications as a composite endpoint including phrenic nerve paralysis, cerebrovascular accident, transient ischemic attacks (TIA), cardiac perforations, pericardial tamponade, pericardial effusion, and death. Effectiveness endpoints included complete PV isolation rate, total procedure time, PVI time, and radiofrequency ablations time to complete PVI (PVI RF time). Notably, total procedure time was not extracted from studies that conducted both classical PVI and other ablations but without a clear record for each process.

Data referring study characteristics and endpoints of interest were extracted by two authors independently from included studies. Study characteristics include study design, patient baseline information, catheters used for ablation, indexes that guided ablation procedure like power, time, and contact force.

The quality of included studies was assessed with the Newcastle-Ottawa scale (NOS), and the quality of RCTs was assessed by RoB2 additionally [14]. This study was reported following the Preferred Reporting Items for Systematic Reviews and Meta-Analyses (PRISMA) statement [15]. The protocol for this systematic review and meta-analysis was registered on PROSPERO (CRD42021266106).

### 2.2. Statistical Analysis

Continuous variables were presented as mean and standard deviation (SD) and pooled by the inverse variance method. Median and interquartile range (IQR) were converted into SD for further pooled analysis by estimation with the method presented by Luo et al. and Wan et al. [16, 17]. Dichotomous variables were presented as risk ratios (RR) and pooled by the Mantel-Haenszel method. The 95% confidence interval (CI) was used in both continuous and dichotomous data while two-sided *P* value of <0.05 was considered as statistically significant. The random-effect model instead of the fixed-effect model was applied in all pooled analyses in this study in consideration of the heterogeneity in study design, including patient population, ablation equipment, and power setting.

The Cochran *Q* test and *I*^2^ test were performed to assess the heterogeneity of the pooled effects. Heterogeneity was considered to exist when the *P* value of Cochran *Q* test < 0.10 and *I*^2^ > 50%. Publication biases were assessed with funnel plot, and the symmetric distribution of effect sizes was assessed visually.

Pooled analysis was performed with Review Manager (RevMan, Version 5.4. The Cochrane Collaboration, 2020). Jackknife Sensitivity analyses were performed for each outcome by systematically leaving out each study from pooled analyses to estimate the effect of every single study on overall estimate and track the origin of heterogeneity. Funnel, Egger's test, and meta-regression were performed with Stata (Version 14.0).

## 3. Results

### 3.1. Included Studies and Their Quality Assessment

**Figure 1:**
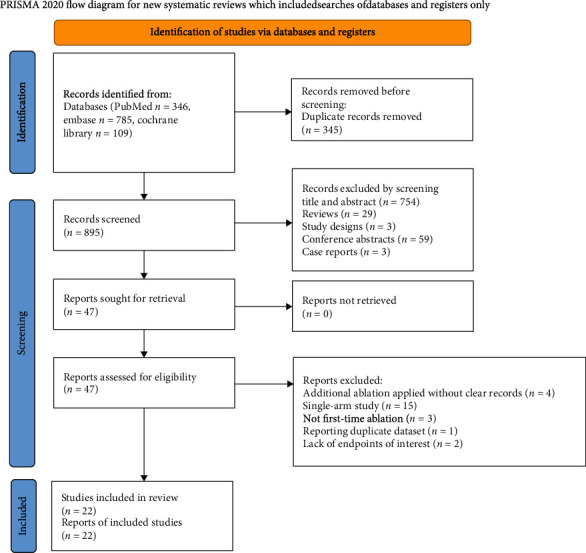
Flow chart demonstration literature screen process.

According to the established searching strategy, 1240 potential relative literatures were identified from PubMed, Embase, and Cochrane Library and from which 22 studies were included in this meta-analysis after full-text review of 47 articles as shown in Figure 1 [7, 8, 18–37]. There were 9 retrospective studies, 10 prospective studies, 1 randomized nonblinded study, and 2 randomized controlled trials (RCTs).

In total, 3867 atrial fibrillation patients were included; among them, 2393 patients received high-power-based RFA. The percentage of paroxysmal AF in all included patients ranged from 39% to 100%. The ablation power setting ranged from 30 W to 90 W in high-power and very-high-power groups and 25 W to 40 W in low-power or conventional groups. Notably, the settings of 20 W to 25 W were also applied in several high-power groups when dealing with pulmonary vein segments adjacent to esophagus. Characteristics of included studies and their radiofrequency ablation procedure-related settings are shown in Tables 1 and 2, respectively. The endpoints of each origin study and their available definitions and the application of antiarrhythmic drugs were shown in Supplementary Table S4.

The quality of included studies was assessed according to NOS from the aspects of selection, comparability, and outcome, as shown in Supplementary Table S5. Moreover, we assessed the risk of bias in three included randomized control trials with RoB2 (Cochrane risk-of-bias tool for randomized trials) from five domains including randomization process, intended interventions, missing outcome data, measurement of the outcome, and selection of the reported result (Supplementary Figure S1).

### 3.2. Results of Meta-Analysis

#### 3.2.1. Efficacy Endpoints of High-Power Short-Duration Ablation Strategies

**Figure 2:**
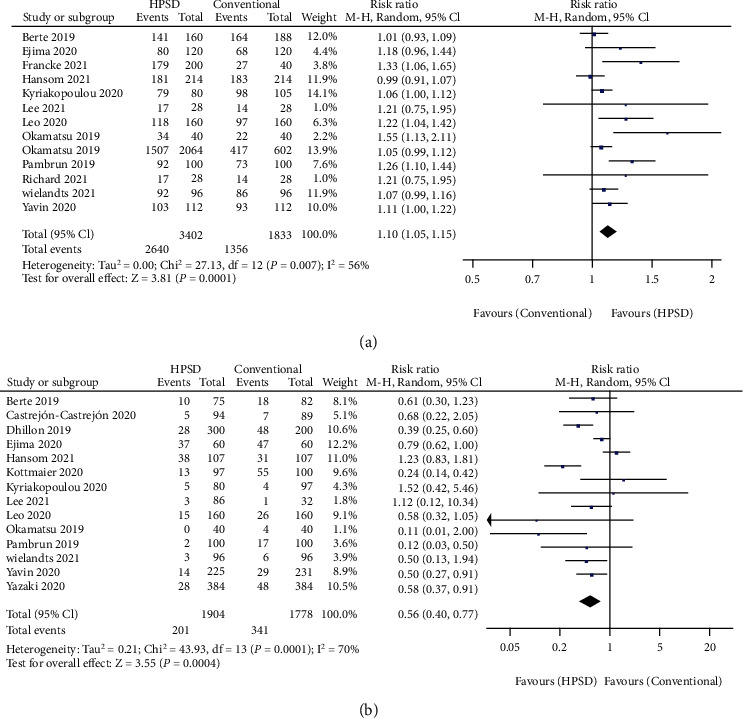
Forest plot of pooled effect demonstrating (a) the first-pass pulmonary vein isolation rate and (b) the acute pulmonary vein reconnection rate of high-power short-duration (HPSD) and conventional ablation settings. 95% CI: 95% confidence interval.

First-pass PVI rate was pooled from 13 studies [19, 21, 24–33, 37]. Patients underwent HPSD ablation showed higher first-pass PVI rate during procedure compared with conventional group (RR = 1.10, 95% CI: 1.05-1.15, *I*^2^ = 56%, and *P* = 0.0001) (Figure 2(a)). Acute reconnection rate during surgery was reported in 14 studies [8, 18, 19, 21, 25, 27–31, 33, 35–37]. More acute PV reconnection during surgery was reported in the conventional setting group compared with the HPSD setting group (RR = 0.56, 95% CI: 0.40-0.77, *I*^2^ = 70%, and *P* = 0.0004) (Figure 2(b)).

**Figure 3:**
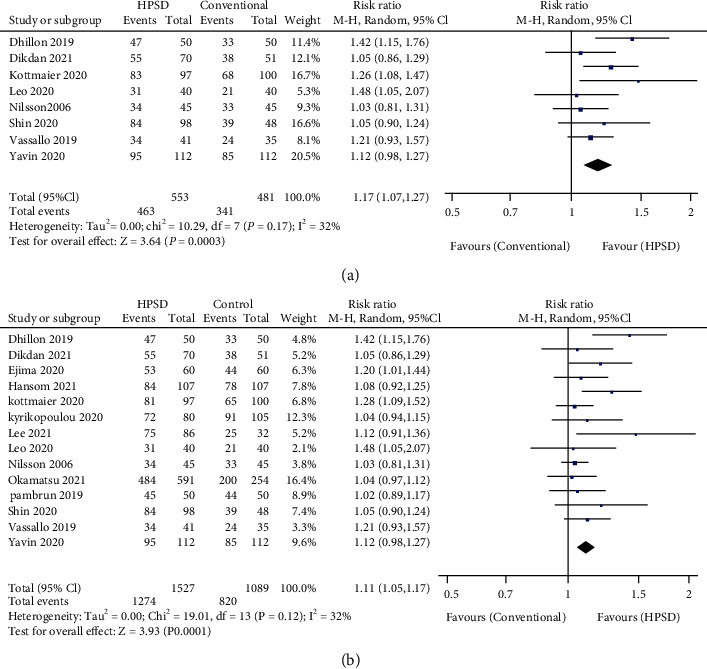
Forest plot of pooled effect demonstrating (a) free from atrial fibrillation (AF) for 12 months and (b) free from atrial tachyarrhythmia (AT) for 12 months in high-power short-duration (HPSD) and conventional ablation settings. 95% CI: 95% confidence interval.

During follow-up, the rates of free from AF and free from AT 6-month and 12-month postsurgery were collected and pooled from 18 studies [7, 8, 19–23, 25–31, 33–35, 37]. Six months after procedure, neither the rate of free from AF nor free from AT reached statistically significant difference between HPSD groups and conventional groups (for free from AF, RR = 1.11, 95% CI: 0.96-1.28, *I*^2^ = 45%, and *P* = 0.16. For free from AT, RR = 1.06, 95% CI: 0.96-1.17, *I*^2^ = 30%, and *P* = 0.24) (Supplementary Figure S2a and S2b). When the follow-up period was elongated to 12 months, HPSD groups showed both higher free from AF and free from AT rates compared with conventional groups (for free from AF, RR = 1.17, 95% CI: 1.07-1.27, *I*^2^ = 32%, and *P* = 0.0003. For free from AT, RR = 1.11, 95% CI: 1.05-1.17, *I*^2^ = 32%, and *P* < 0.0001) (Figures 3(a) and 3(b)).

We performed subgroup analyses for 12 months free from AF and AT according to the ablation power setting and whether the ablation was guided by integral indexes or not. Either when the ablation power was set ≤45 W or between 45 W and 60 W for left atrial walls except for posterior walls, HPSD groups showed better free from AF rate (for power ≤ 45 W, RR = 1.28, 95% CI: 1.02-1.61, *I*^2^ = 58%, and *P* = 0.03; for 45 W < power < 60 W, RR = 1.10, 95% CI: 1.01-1.19, *I*^2^ = 0%, and *P* = 0.03, Supplementary Figure S3a). Although RR did not achieve a statistically significant difference between the two subgroups (*P* = 0.21), the 45 W < power < 60 W subgroup did not show heterogeneity inside the group while the other group remained high heterogeneity. Higher free from AT rate was also observed in both subgroups (for power ≤ 45 W, RR = 1.17, 95% CI: 1.01-1.36, *I*^2^ = 63%, and *P* = 0.04; for 45 W < power < 60 W, RR = 1.09, 95% CI: 1.04-1.14, *I*^2^ = 3%, and *P* = 0.0006, Supplementary Figure S3b) compared with conventional groups. Similarly, the 45 W < power < 60 W subgroup showed minor heterogeneity while the ≤45 W group remained high heterogeneity.

Subgroup analyses for free from AF or AT 12-month after surgery were also performed according to whether ablations were guided by integral indexes including force-time integral (FTI), AI, and LSI or with fixed ablation time per point. HPSD groups always showed better outcomes of free from AF (for integral indexes guided, RR = 1.28, 95% CI: 1.02-1.60, *I*^2^ = 61%, and *P* = 0.03; for fixed ablation time, RR = 1.13, 95% CI: 1.07-1.27, *I*^2^ = 0%, and *P* = 0.001. Supplementary Figure S4a) while the AF free rate did not differ between two subgroups (*P* = 0.31). HPSD also showed better free from AT rate in both subgroups (for integral indexes guided, RR = 1.11, 95% CI: 1.02-1.20, *I*^2^ = 49%, and *P* = 0.01; for fixed ablation time, RR = 1.12, 95% CI: 1.05-1.19, *I*^2^ = 7%, and *P* = 0.0009. Supplementary Figure S4b) compared with conventional groups while the AT free rated between two subgroups did not achieve statistically significant difference *P* = 0.87).

Additional subgroup analyses according to the application of antiarrhythmic drugs (AADs) postsurgery after the three-month blank period were performed because the status of AADs could be another source of heterogeneity. Since the length of blank period differed in origin studies, we set the longest blank period among included studies, 3 months, as our defined blank period. The HPSD group showed higher 12 months free from AF rate in subgroups without AADs after blank period (RR = 1.27, 95% CI: 1.08-1.49, *I*^2^ = 0%, and *P* = 0.004) and with unclear AAD status (RR = 1.15, 95% CI: 1.05-1.26, *I*^2^ = 7%, and *P* = 0.004). However, the free from AF rate did not show statistically significant difference between the HPSD and the conventional groups in the subgroup with unknown AAD status after the blank period (RR = 1.05, 95% CI: 0.92-1.20, *I*^2^ = 0%, and *P* = 0.49). All subgroups showed low heterogeneity inside each group, and the pooled effects did not show difference among subgroups (*P* = 0.19) (Supplementary Figure S5a). The free from AF or AT rate 12 months postsurgery showed a similar trend. The HPSD group had higher 12 months free from AF or AT rate in subgroups without ADDs or with unclear ADD status after blank period (for subgroup without AADs, RR = 1.17, 95% CI: 1.07-1.27, *I*^2^ = 24%, and *P* = 0.0004; for subgroup with unknown AAD status, RR = 1.06, 95% CI: 1.00-1.13, *I*^2^ = 0%, and *P* = 0.04). In the subgroup with continued AADs after the blank period, the difference of 12 months free from AF or AT rate between HPSD and conventional groups did not reach statistical significance (RR = 1.05, 95% CI: 0.92-1.20, *I*^2^ = 0%, and *P* = 0.49). The heterogeneity inside each subgroup was low, and the pooled effects did not show difference among subgroups (*P* = 0.17) (Supplementary Figure S5b).

#### 3.2.2. Safety of High-Power Short-Duration Ablation Strategies

**Figure 4:**
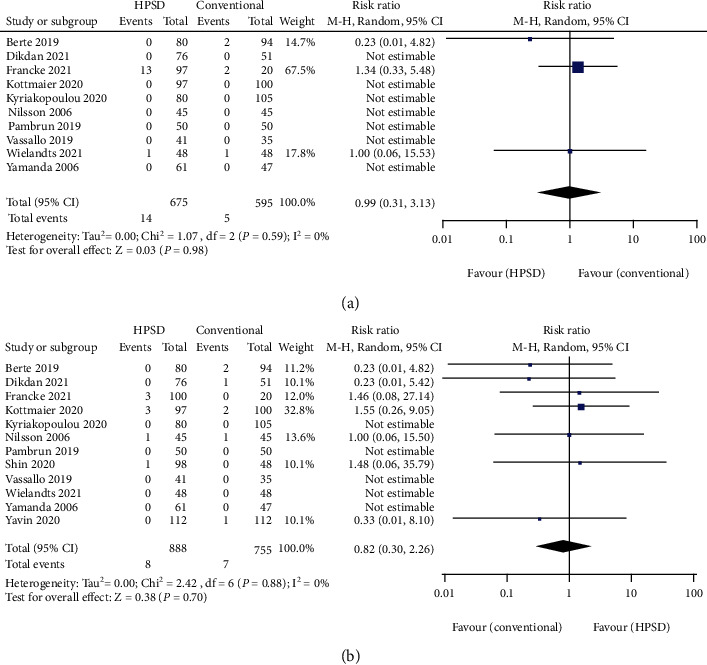
Forest plot of pooled effect demonstrating (a) esophageal injury and (b) major complications in high-power short-duration (HPSD) and conventional ablation settings. 95% CI: 95% confidence interval.

Esophageal injuries correlated with PVI RFA occurred in 3 studies [21, 32, 37], while other 7 studies did not report any PVI-related esophageal injury during or after surgery [7, 8, 20, 22, 25, 30, 34]. The overall effect did not show statistically differences between HPSD and conventional ablation setting groups on esophageal injuries (RR = 0.99, 95% CI: 0.31-3.13, *I*^2^ = 0%, and *P* = 0.98) (Figure 4(a)).

Major complications other than esophageal injury were reported in 12 studies [7, 8, 19–23, 25, 30, 32, 35, 37]. RFA-related major complications in this study were defined as phrenic nerve paralysis, CVA, TIA, cardiac perforations, pericardial tamponade, pericardial effusion, and death during periprocedural period and were clearly attributed to the radiofrequency ablation. Thus, 1 case with comorbidity of pericardial effusion existed at baseline was excluded from Yavin's study in this analysis [18]. Vascular complications like groin hematoma were excluded since the hematoma formation was irrelevant to ablation itself but with femoral venous puncture. Among 12 included studies, we did not detect statistically significant difference of major complications between the HPSD group and the conventional group (RR = 0.82, 95% CI: 0.30-2.26, *I*^2^ = 0%, and *P* = 0.70) (Figure 4(b)).

#### 3.2.3. Ablation Effectiveness of High-Power Short-Duration Ablation Strategies

**Figure 5:**
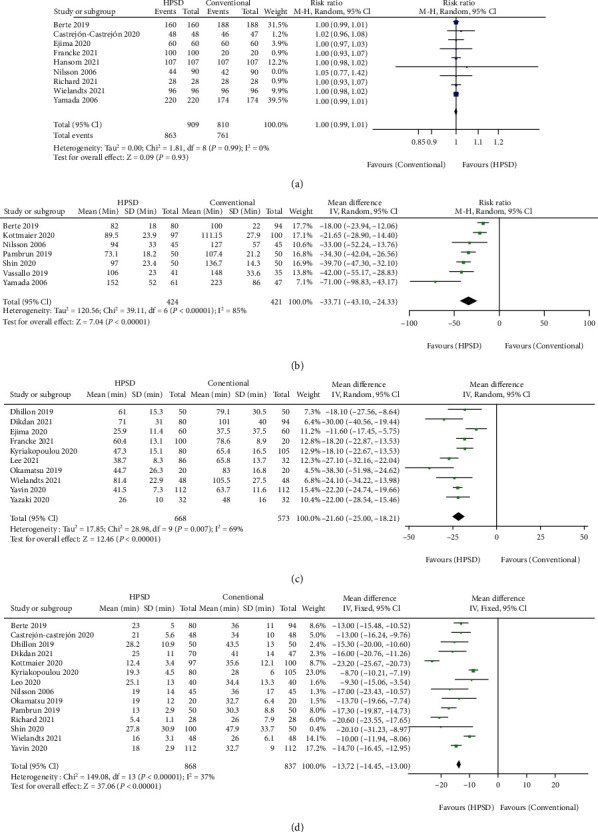
Forest plot of pooled effect demonstrating (a) complete pulmonary vein isolation rate, (b) total procedure time, (c) pulmonary vein isolation (PVI) time, and (d) radiofrequency ablation applied time for PVI of high-power short-duration (HPSD) and conventional ablation settings. 95% CI: 95% confidence interval.

The rate of complete PVI was pooled from 9 studies, and there was no statistically significant difference between HPSD and conventional groups on successfully complete PVI (RR = 1.00, 95% CI: 0.99-1.01, *I*^2^ = 0%, and *P* = 0.93) (Figure 5(a)) [7, 20, 21, 24, 31–33, 36, 37]. We only performed pooled analysis on total procedure time in 7 studies because of additional ablations were widely applied but were not recorded or presented separately, like roof linear ablation, LA posterior wall box isolation, superior vena cava (SVC) isolation, and cavotricuspid isthmus ablation [7, 8, 20, 22, 23, 25, 37]. The HPSD group showed significant shorter total procedure time compared with the conventional group (MD = −33.71 95% CI: -43.10 to -24.33, *I*^2^ = 85%, and *P* < 0.00001) (Figure 5(b)). PVI time was reported in 10 studies [18, 19, 21, 27, 29, 30, 32–35]. PVI time was shorter in the HPSD group than the conventional group (MD = −21.60 95% CI: -25.00 to -18.21, *I*^2^ = 79%, and *P* < 0.00001) (Figure 5(c)). The RFA time during PVI procedure (PVI RF time) was reported in 14 studies [7, 8, 19, 21, 23–25, 27, 28, 30, 34–37]. Coincide with total procedure time, it took the HPSD group significantly shorter PVI RF time than the conventional group (MD = −13.72, 95% CI: -14.45 to -13.00, *I*^2^ = 91%, and *P* < 0.00001) (Figure 5(d)). Subgroup analyses were performed according to the power setting as mentioned above to seek the source of the heterogeneity.

The 45 W < power < 60 W subgroup showed significant shorter PVI RF time compared with the ≤45 W subgroup (*P* < 0.00001). Nevertheless, the 45 W < power < 60 W subgroup had minor heterogeneity (MD = −15.60, 95% CI: -16.91 to -14.29, *I*^2^ = 0%, and *P* < 0.00001) while the ≤45 W subgroup remained high heterogeneity (MD = −10.49, 95% CI: -13.13 to -8.75, *I*^2^ = 70%, and *P* < 0.00001, Supplementary Figure S6).

### 3.3. Publication Bias

Funnel plots were applied to outcomes pooled from more than 9 studies, and symmetrical distribution of the funnel plot was measured with Egger's test. Acute PV reconnection (*P* = 0.247), esophageal injury (*P* = 0.402), major complications (*P* = 0.136), PVI time (*P* = 0.602), and PVI RF time (*P* = 0.246) were considered not having publication bias while the publication bias existed in first-pass PVI (*P* = 0.007) and free from AT 12 months after surgery (*P* = 0.014) (Supplementary Figures S7-S13) [38, 39].

### 3.4. Metaregression

Both observational studies and RCTs were included in this meta-analysis. To investigate whether the type of study introduced heterogeneity into pooled effects, we performed metaregression by defining the study design as the independent variable effect size of each outcome as the dependent variable. As shown in Supplementary Table S6, the independent variable would not significantly affect the effect size (*P* > 0.05 in all endpoints), indicating that it is rational to pool data from included observational studies and RCTs.

## 4. Discussion

This study is an updated meta-analysis on the efficacy, safety, and effectiveness of HPSD vs. conventional strategies in AF patients, and more importantly, the first meta-analysis made effort on distinguishing different RFA procedures of AF and focused on the classic and effective PVI procedure. We found that HPSD RFA for AF patients showed better efficacy compared with conventional group, reflected in higher first-pass PVI rate, less acute PV reconnection rate, and higher free from AF or AT rate 12 months postsurgery. This study proved a promising safety profile for HPSD setting since there was no difference between esophagus injury or other major complications between HPSD and conventional setting groups. From the aspect of effectiveness, the HPSD group showed significantly reduced total procedure time, PVI time, and PVI RF time while the rate of complete PVI did not differ between HPSD and conventional groups.

PVI has been a fundamental RFA technique for AF. However, operators may choose additional ablations on non-PV targets based on the characteristic of each patient. For instance, cavotricuspid isthmus ablation has been a well-established ablation strategy for typical atrial flutter and was typically applied to patients who have comorbidity of atrial flutter, while box isolation has become a new attempt dealing with permanent atrial fibrillation as a complement of PVI [40–42]. These abovementioned additional ablations reflected the heterology of each patient and introduced different extra procedure time, left atrial dwelling time, and radiofrequency time to each patient's procedure and consequent different recurrent rate of AF or AT during follow-up. Therefore, these data should not be pooled with the clear existence of the procedure heterology. Studies included additional ablations reported that HPSD groups had superior effectiveness and parallel safety profile to conventional groups which coincided with our pooled results, while the efficacy outcomes including recurrence of atrial tachycardia were disparate. Baher et al. reported that HPSD (50 W/5 s) ablation resulted in shorter procedure time, similar esophagus injury pattern, and similar long-term AF recurrence rate [43]. Bunch et al. reported HPSD group was associated with reduced procedure times, similar free from AF rate but increased rate of recurrent atrial flutter [44]. Counting the heterology and potential bias would be brought by additional ablations, and some studies though with large patients scales were excluded in this analysis if the data of additional ablations was not reported. To further evaluate the efficacy, safety, and effectiveness of HPSD-based radiofrequency ablation, more studies including rigorous design, grouping, and recording are needed.

Radiofrequency ablation, by forming permanent transmural heating lesions in left atrium tissue, blocks the ectopic current transduction to attenuate atrial fibrillation generation. During radiofrequency ablation, durable lesion formation is critical for long-term effectiveness. Power, as the parameter that impacts lesion characteristics and can be easily manipulated, has become one of the research interests to improve the quality of radiofrequency ablation. Bourier et al. demonstrated with in silico model that HPSD ablation (50 W/11 s to 13 s) had produced the equivalent lesion compared with conventional setting (30 W/30s) [9]. Another in silico model showed that higher power and shorter duration ablation settings were correlated with wider but shallower depth of lesions [45]. With an in vitro experiment, Bhaskaran et al. reported that HPSD RFA (50 W/5 s or 60 W/5 s) induced comparable lesion width and depth on myocardial phantom compared with standard-setting (40 W/30 s), while higher power setting (70 W/5 s and 80 W/5 s) resulted in larger lesion width [46]. Yavin et al. further proved with swine's atria that HPSD ablation (90 W/4 s) created shallower but wider lesions on ablation sites [19]. All literature mentioned above considered that higher power indeed was correlated with shallow while wide lesions with certain radiofrequency ablation settings. Therefore, HPSD setting could be a feasible and more efficient radiofrequency ablation choice in AF ablation theoretically and was supported by our pooled analysis which did not show difference in complete pulmonary vein isolation between the HPSD and conventional groups. Better efficacy outcomes were also proved by this meta-analysis; in specific, the HPSD group showed a higher first-pass PVI rate, less acute PV reconnection, and a higher rate of free from AF 12 months postprocedure. With subgroup analyses, when power ≤ 45 W or 45 W < power < 60 W, HPSD groups both showed better outcomes of 12 months free from AF or AT. Moreover, heterogeneity was minimized in the 45 W < power < 60 W subgroup in both free from AF and AT outcomes, which indicated that ablation power ≤ 45 W in the HPSD group was the source of heterogeneity. The prescription of ADDs may also introduce heterogeneity between studies so we performed subgroup analyses according to the status of ADDs application. The HPSD groups presented higher free from AF and free from AT 12-month postsurgery in subgroups both with discontinued ADDs after the blank period and subgroups with unclear ADDs status. However, in the subgroup with continued ADDs, the HPSD strategy did not show superiority over the conventional group in 12 months free from AF or AT, which may indicate that the better efficacy of the HPSD strategy can be partially complemented by ADDs. Nevertheless, selection bias brought by the different severity of AF, type of AF, etc., in origin studies may also cause this comparable result. Whether the superiority of HPSD efficacy is correlated with the shallow and wide lesion or other special characteristics, the reason HPSD creates more durable lesions requires further study. Very-high-power short duration radiofrequency ablation setting had become another attempt to improve ablation outcomes by altering the power setting recently but with limited research [8, 24]. Further studies are necessary to understand the efficacy, safety, and effectiveness of higher power setting during radiofrequency ablation.

Durable lesion formation is critical for long term free from AF after radiofrequency ablation. Several integral parameters cooperating factors that matter for high-quality lesions such as FTI, ablation index (AI), lesion size index (LSI), and CLOSE protocol have been introduced into practice to improve the quality of lesions. FTI was the initial attempt to combine CF, and ablation and has been proven correlated with transmurality of RFA lesions [47]. FTI was later replaced by AI and other indexes attributed to several flaws like not counting power and the assumption of the linear relationship between CF and time. AI, a weighted nonlinear formula of contact force, power, and ablation duration, has been proven more favorable than FTI by providing more information and was able to predict PV connection [5, 48]. Lesion size index (LSI), another index calculated by formula of time, power, CF, and impedance, was reported a better predictor for ablation lesion dimensions than power or CF only [3, 4]. We performed subgroup analyses according to whether integral indexes or used during the procedure or not and found that either integral index-guided ablation or time-fixed ablation resulted in better free from AF or AT outcomes 12 months postsurgery. Integral index-guided ablation subgroups seemed to show a more potent outcome of free from AF than time fixed subgroup though not reached statistically significant yet. Integral index-guided ablation showed high heterogeneity while time-fixed ablation did not show heterogeneity, which could attribute to pooling different index-guided ablations.

A potential advantage of HPSD radiofrequency ablation for AF is the procedure safety, especially from the prospection of esophagus thermal injury. A segment of esophagus is in direct contact with posterior wall of left atrium, and the atria portions adjacent to esophagus differed from most pulmonary vein openings to the left atrial antrum. Corresponding to the relative orientation between esophagus and left atrial, the distance from esophagus wall to endocardium range from 3.3 to 13.5 mm while the tissue thickness between pulmonary vein ostia and esophagus ranged from 7.7 to 32.8 mm [49, 50]. Esophagus injury has been a spectrum of devastating complications during AF ablation. Being thinner than most parts of left atrial wall and situated nearby esophagus, ablation performed on posterior wall made the esophagus more prone to thermal injury so radiofrequency ablation has a narrow efficacy and safety window [51]. Our pooled study did not show differences in esophagus injury between HPSD and conventional groups. However, when taking the severity or classification of individual esophagus injury into account, HPSD strategy seems to show a trend of superiority. Francke et al. reported 2 of 20 (10%) and 13 of 97 (13.4%) thermal esophageal lesions in standard (conventional, 20-40 W/AI: 400-500) or HPSD (50 W/AI: 400-500) groups, respectively (*P* = 0.72). Both lesions in the standard group were deep ulcers and resolved slowly in 2 weeks while most lesions in the HPSD group were smaller and more superficial with only one patient developed a large ulcer [32]. Leo et al. monitored esophagus temperature with a probe and recorded statistically significant increased esophagus temperature alert number per patient in the low-power group (20 W/LSI = 4 or 5) compared with high-power (40 W/LSI = 4 or 5) groups (*P* = 0.026) [28]. Opposite evidence was reported as well, for example, Yavin et al. reported a higher temperature recorded in the high-power group (45-50 W/8 or 15 s) as 39.2 ± 2.2°C compared with the medium-power group (20-40 W/20 to 30s) as 38.1 ± 1.1°C (*P* = 0.032) [19]. Notably, operators usually choose to lower the power when handling structures located on the left atrial posterior wall for safety reasons. For example, Leo et al. applied 20 W at the posterior part of the pulmonary vein during PVI for both HPSD and conventional groups, and Lee et al. chose 25 W at posterior wall for both groups [28, 29]. In other included studies, the power applied to posterior wall was also reduced compared with other locations in the antrum. These lowered power ablations at posterior wall could contribute to the comparative rate of esophagus thermal injury rate between HPSD and conventional groups. However, this attempt also made the safety profile of HPSD ablation at posterior wall sites remain unclear. As mentioned before, HPSD radiofrequency creates shallower lesions in silico and in vivo, which could theoretically lessen the thermal injury rate during the procedure. Whether a higher power setting is truly safe for esophagus still requires studies carefully coordinating the distance between endocardium and esophagus, the power applied during ablation, and the esophagus injury patterns in the future.

In this study, the HPSD group showed better effectiveness over the conventional group, including total procedure time, PVI time, and PVI RF time. Shortening the procedure time, especially for those patients who underwent only local anaesthesia instead general anaesthesia, helps comfort patients and therefore improves their adherence. With reduced PVI time and PVI RF time, less saline would be irrigated into circulation and may mitigate the burden of heart pump brought by overwhelmed liquid. Shorter fluoroscopic time was also reported in several studies though we did not include these data because it was hard to tell whether the fluoroscopic time was consumed by PVI or additional ablations [8, 23, 30]. In any case, compressing the fluoroscopic time benefits both patient and operator by reducing radiation exposure no matter the ablation type.

HPSD and very high-power short-duration (vHPSD) strategies have shown satisfying efficacy, safety, and effectiveness in radiofrequency ablation treatment for AF patients in this meta-analysis. Theoretically, HPSD and vHPSD strategies can also be applied in other radiofrequency ablation treatments aiming at arrhythmia like atrial flutter, atrial tachycardia, and premature ventricular contraction. Recently, a case of successful very high-power short-duration radiofrequency ablation without periprocedural complications for frequent premature ventricular contraction with was reported [52]. Whether HPSD or vHPSD is superior to conventional radiofrequency ablation strategies requires further studies.

Although the HPSD strategy brought cardiologists a more efficient and safer way to perform transcatheter radiofrequency ablation, one should always keep in mind that AF clinical management is complex and more than ablation. Radiofrequency ablation treatment serves as rhythm control to achieve better symptom control, while anticoagulation remains the dominant therapy that lessens the thromboembolic stroke risk [53, 54]. The decision of whether continue anticoagulation therapy postablation or not should be made based on stroke risk factors, e.g. CHA_2_DS_2_-VASc, instead of the AF recurrence status [54].

There were several limitations in this study. Firstly, all the studies included were nonrandomized observational studies except for only two RCTs and one randomized nonblinded study, which made the results need to be interpreted and translated cautiously. More RCTs focus on the relationship between radiofrequency power and procedure efficacy and safety should be expected and will provide more persuasive evidence. Secondary, the power settings, ablation time, contact force, AI settings, LSI settings, and the experience of individual operators differed between included studies, and the pooled analysis was not adjusted by the above variables. Although subgroup analysis is based on whether guided by AI or not and the power settings, these categorizing algorithms were still not precise enough to eliminate the heterogeneity. Thirdly, as emphasized multiple times above, the existence of additional ablation targets other than PVI impeded comprehensive analysis of the effect of power setting on AF radiofrequency treatment outcomes. Though we tried to subtract additional ablation-related procedure time or PVI time when there were detailed records and got a decent quantity of studies included for procedure parameter outcomes, the studies qualified for efficacy outcomes, i.e., free from AF and/or AT, were still limited. Future studies with more meticulous recording and analysis for each ablation target and their potential related efficacy and safety outcomes will be demanded to understand how power setting affects procedure outcomes. Last but not the least, publication bias existed in several outcomes, which may be caused by difficulties in publishing negative results. The potential publication results also require careful interpretation of corresponding results.

## 5. Conclusion

The high-power short-duration strategy showed better radiofrequency ablation procedure efficacy and effectiveness compared with the conventional ablation setting. Moreover, HPSD strategy is a safe approach with a similar complication rate for AF radiofrequency ablation procedure.

## Figures and Tables

**(a):** 

Study ID (author, year)	Country	Treatment group	Patients (*n*)	Male (*n*, %)	Age (Y)	Paroxysmal AF (*n*, %)	CHA_2_DS_2_-VASCs	LAD (mm) or size	Follow-up period (month)	Study design	NOS
Nilsson, 2006 [7]	Denmark	HPSD	45	15 (33)	55 ± 10	26 (58)	N/A	N/A	15 ± 7 (range 5–25 months)	Retrospective	8
		LPLD	45	36 (80)	51 ± 11	21 (47)	N/A	N/A			
Yamada, 2006 [20]	Japan	HPSD	61	51 (84)	59 ± 10	61 (100)	N/A	35 ± 6	6	Retrospective	8
		LPLD	47	40 (85)	56 ± 14	47 (100)	N/A	35 ± 4			
Dhillon, 2019 [35]	United Kingdom	HPAI	50	35 (70)	N/A	50 (100)	N/A	N/A	12	Prospective	7
		Conventional	50	24 (48)	N/A	50 (100)	N/A	N/A			
Berte, 2019 [37]	Switzerland	HPSD-CLOSE	80	50 (63)	62 ± 9	65 (81)	N/A	N/A	6	Prospective	8
		Standard-CLOSE	94	67 (71)	63 ± 9	74 (79)	N/A	N/A			
Okamatsu, 2019 [27]	Japan	HP	20	13 (65)	65 ± 10	13 (65)	2 (1-3)	40 ± 6	6.5 (6.3-6.7)	Prospective observational	7
		LP	20	15 (75)	68 ± 8	16 (80)	2 (1-2)	39 ± 6			
Pambrun, 2019 [25]	French	HPSD	50	35 (70)	65 ± 8.2	50 (100)	N/A	107.6 ± 23.1 mL^a^	12	Prospective	9
		LPLD	50	30 (60)	62.5 ± 10.6	50 (100)	N/A	102.9 ± 20.1 mL^a^			
Vassallo, 2019 [22]	Brazel	HPSD	41	34 (83)	64 ± 10	28 (68)	2 ± 1.6	43.3 (28-62)	12	Retrospective	9
		Conventional	35	22 (63)	61 ± 12	27 (77)	2 ± 1.97	41.9 (23-56)			
Castrejón-Castrejón, 2020 [36]	Spain	HPSD	48	32 (67)	61 ± 10	31 (65)	N/A	N/A	3	Retrospective	6
		Conventional	47	28 (60)	60 ± 10	30 (64)	N/A	N/A			
Ejima, 2020 [33]	Japan	HPSD	60	44 (73)	63 ± 11.3	60 (100)	1.8 ± 1.4	34.3 ± 10.3	20.7 ± 2.0	Prospective cohort	8
		Conventional	60	42 (70)	66.7 ± 8.9	60 (100)	2.2 ± 1.4	36.1 ± 8.7			
Kottmaier, 2020 [8]	Germany	VHPSD	97	57 (59)	60.8 ± 13.9	97 (100)	1.95	N/A	12	Prospective	9
		LPLD	100	60 (60)	60.8 ± 10.5	100 (100)	1.64	N/A			
Kyriakopoulou, 2020 [30]	Belgium	HPSD	80	47 (59)	67 (58-73)	80 (100)	2 (1-3)	43 ± 8	12	Retrospective	8
		LPLD	105	65 (62)	64 (56-69)	105 (100)	2 (1-2)	44 ± 6			
Leo, 2020 [28]	United Kingdom	HPSD	40	26 (65)	60.7 ± 9.4	16 (40)	N/A	42.6 ± 8	29.4 ± 3.9	Prospective, randomized, unblinded	8
		LPLD	40	33 (83)	57.3 ± 9.6	15 (38)	N/A	42.7 ± 6.8			
Shin, 2020 [23]	Korea	HPSD	100	81 (81)	57.9 ± 9.4	48 (48)	1.65 ± 1.4	40.7 ± 5.5	12	Prospective randomized controlled trial	7
		LPLD	50	33 (66)	58.7 ± 11.1	24 (48)	1.7 ± 1.6	40.7 ± 6.5			
Yavin, 2020 [19]	USA	HPSD	112	71 (63)	62.3 ± 5.2	76 (68)	2.4 ± 1.3	44.2 ± 4.7	14.2 (1.9-35)	Prospective	7
		MPLD	112	79 (71)	64.8 ± 7.2	67 (60)	2.6 ± 1.4	47.1 ± 5.1	22.8 (3-43.9)		
Yazaki, 2020 [18]	Japan	HPSD	32	27 (84)	61 ± 11	22 (69)	N/A	40 ± 13	10 (4–12)	Retrospective	7
		LPLD	32	20 (63)	66 ± 11	29 (91)	N/A	41 ± 14			
Dikdan, 2021 [34]	USA	HPSD	76	54 (71)	63.2 ± 11.1	32 (42)	2 (1-3)	145.4 ± 49.9 mL^a^	12	Retrospective	9
		SPSD	51	40 (78)	60.7 ± 9.8	21 (41)	1 (1-2)	162.3 ± 39.9 mL^a^			
Wielandts, 2021 [21]	Belgium	HP-CLOSE	48	32 (67)	64 ± 11	48 (100)	1 (0-3)	39 ± 7	6	Prospective randomized controlled	8
		LP-CLOSE	48	33 (69)	61 ± 11	48 (100)	1 (0-3)	40 ± 7			

**(b):** 

Study ID (author, year)	Country	Treatment group	Patients (*n*)	Male (*n*, %)	Age (Y)	Paroxysmal AF (*n*, %)	CHA_2_DS_2_-VASCs	LAD (mm) or size	Follow-up period (month)	Type of study	NOS
Francke, 2021 [32]	Germany	HP-CLOSE	100	60 (60)	66.4 ± 10	49 (49)	2.8 ± 1.5	N/A		Prospective trial	8
		Standard-CLOSE	20	7 (35)	66.4 ± 10	9 (45)	3.2 ± 1.5	N/A	3		
Hansom, 2021 [31]	Canada	HPSD	107	69 (64)	62 ± 9	67 (63)	1.9	41 ± 0.7	12	Retrospective cohort	8
		FTI-guided LDLD	107	81 (76)	62 ± 9	60 (56)	2	41 ± 0.6			
Richard, 2021 [24]	Germany	VHPSD	28	21 (75)	69 (61-73)	11 (39)	N/A	26 (25-35)^b^	N/A	Prospective	7
		Conventional	28	19 (68)	69 (62-75)	14 (50)	N/A	32 (26-39)^b^			
Lee, 2021	Korea	HPAI	86	66 (77)	59.5 ± 9	62 (72)	1 (1-2)	42.6 ± 4.5	12	Prospective	8
		CPAI	32	25 (78)	59.9 ± 9.1	24 (75)	1 (0-2)	41.2 ± 5.4			
Okamatsu, 2021 [26]	Japan	HP	1032	716 (69)	68 (61-74)	583 (56)	2 (1-3)	41 (37-46)	12	Retrospective	8
		CP	301	210 (70)	67 (61-73)	172 (57)	2 (1-3)	41 (37-46)			

Values are presented as mean ± SD, medians (interquartile range), or *n* (%). ^a^The left atrium size was measured by left atrial volume in mL; ^b^the left atrial size was presented with the unit of ml/m^2^ per body surface area. BMI: body mass index; CHA2DS2-VASCs: 1 point for each of congestive heart failure, hypertension, diabetes, vascular disease, age of 65-74 years old or female, 2 points for each of stroke or age ≥ 75 years old; LAD: left atrium diameter; LVEF: left ventricular eject fraction; HPSD: high-power short-duration; LPLD: low-power long-duration; HPAI: high-power ablation index- (AI-) guided; CLOSE: CLOSE protocol; HP: high-power; LP: low-power; VHPSD: very-high-power short-duration; MPLD: moderate-power moderate-duration; CPAI: conventional-power AI-guided.

**(a):** 

Study ID (author, year)	Mapping system	Ablation catheter	Treatment group	Power (W)	Ablation time per point (s)	AI or LSI	Contact force setting (g)
Nilsson, 2006 [7]	LASSO	Celsius ThermoCool	HPSD	Max = 45	20	N/A	N/A
			LPLD	Max = 30	120		
Yamada, 2006 [20]	QMS2™	Constellation	HPSD	Max = 40	60	N/A	N/A
			LPLD	Max = 30	60		
Dhillon, 2019 [35]	CARTO	Thermocool STSF or ST	HPAI	30 (posterior), 40 (elsewhere)^a^	N/A	AI: 350-450	10-20
			Conventional	25 (posterior), 30 (elsewhere)^a^			
Berte, 2019 [37]	LASSO NAV	ThermoCool STSF	HPSD-CLOSE	35 (posterior), 45 (elsewhere)^a^	N/A	AI: ≥400, (posterior) ≥550 (anterior)^a^	N/A
			Standard-CLOSE	25 (posterior), 35 (elsewhere)^a^			
Okamatsu, 2019 [27]	CARTO-3	ThermoCool STSF	HP	40 (posterior), 50 (elsewhere)^a^	N/A	AI: 400 (anterior), 360 (posterior) 260 (esophagus)^a^	N/A
			LP	20 (posterior), 30 (elsewhere)^a^			
Pambrun, 2019 [25]	CARTO-3	ThermoCool ST	HPSD	40 (posterior), 50 (elsewhere)^a^	8.5 ± 0.8	N/A	N/A
			LPLD	25 (posterior), 30 (elsewhere)^a^	15.7 ± 2.3		
Vassallo, 2019 [22]	Ensite velocity 4.0 or 5.0	TactiCath	HPSD	45 (posterior), 50 (elsewhere)^a^	6	N/A	8-20
			Conventional	30	30		10-30
Castrejón-Castrejón, 2020 [36]	CARTO-3	TactiCath™ Quartz and SE, Abbott, or ThermoCool ST	HPSD	50-60	7 or <30	LSI > 5 or AI > 350 (posterior) AI > 450 (elsewhere)^a^	5-40
			Conventional	20-30	30-60	N/A	N/A
Ejima, 2020 [33]	CARTO	ThermoCool STSF	HPSD	50	3-5	N/A	5-20
			Conventional	25 (esophagus), 30-40 (elsewhere)^a^	5-10		10-20
Kottmaier, 2020 [8]	N/A	Abbott flexibility SE catheter	VHPSD	70	5-7	N/A	N/A
			LPLD	30-40	20-40		
Kyriakopoulou, 2020 [30]	LASSO	ThermoCool ST	HPSD	40	20-40	AI: ≥400 (posterior/roof/south pole), ≥ 550 (anterior)^a^	12-15
			LPLD	35	N/A		12-15
Leo, 2020 [28]	Ensite velocity, precision or Optima	TactiCath Quartz	HPSD	20 (posterior), 40 (elsewhere)^a^	N/A	LSI: 6 (anterior), 4 or 5 (posterior)^a^	Min = 10
			LPLD	20			
Shin, 2020 [23]	CARTO-3	ThermoCool STSF	HPSD	25-30 (posterior), 40 or 50 (elsewhere)^a^	10 or 20	N/A	Blind to operators
			LPLD	25-30 (posterior), 30 (elsewhere)^a^	40		
Yavin, 2020 [19]	CARTO-3	ThermoCool STSF	HPSD	45-50	8 (posterior), 15 (elsewhere)^a^	N/A	N/A
			MPLD	20 (posterior), 30-40 (elsewhere)^a^	20 (posterior), 30 (elsewhere)^a^		

**(b):** 

Study ID (author, year)	Mapping system	Ablation catheter	Treatment group	Power (W)	Ablation time per point (s)	AI, LSI, or FTI	Contact force (g)
Yazaki, 2020 [18]	CARTO-3	ThermoCool	HPSD	50	8-12	N/A	5-15
			LPLD	20-25 (posterior), 25-40 (elsewhere)^a^	30		10-20
Dikdan, 2021 [34]	St. Jude EnSite Velocity system	ThermoCool	HPSD	50	15	LSI: 5 (posterior) 6 (elsewhere)^a^	8-40
			SPSD	20-25	30-60	LSI: 4.5–5.5	10-40
Wielandts, 2021 [21]	N/A	ThermoCool ST	HP-CLOSE	45	N/A	AI: ≥550 (anterior), ≥400 (elsewhere)^a^	≤30
			LP-CLOSE	35			
Francke, 2021 [32]	Carto 3	ThermoCool STSF	HP-CLOSE	50	N/A	AI: 400 (posterior) and 550 (anterior)^a^	N/A
			Standard-CLOSE	20 (posterior), 40 (elsewhere)^a^			
Hansom, 2021 [31]	Carto 3, PentaRay, or Lasso	ThermoCool	HPSD	50	6-8 (posterior)^a^ 8-10 (elsewhere)^a^	N/A	10-20
			FTI-guided LDLD	20 (posterior), 30 (elsewhere)^a^	Determined by LSI, typically 20-25 (posterior) 30-40 (elsewhere)^a^	300 ≤ FTI ≤ 400 g·s (posterior)^a^ FTI > 400 g·s (elsewhere)^a^	
Richard, 2021 [24]	Carto 3	QDOT Microcatheter	VHPSD	90	4	N/A	N/A
			Conventional	25-40	N/A	AI: 550 (anterior), 450 (roof), 380 (posterior)^a^	10-40
Lee, 2021 [29]	Carto 3	ThermoCool STSF	HPAI	25, 30-40	N/A	AI: ≥450 (anterior/roof), ≥350 (posterior/inferior/carina)^a^	5-20
		ThermoCool ST	CPAI	25-35			
Okamatsu, 2019 [27]	Carto 3	ThermoCool STSF	HP	25, 40-50	N/A	AI: ≥400 (anterior), ≥360 (posterior), ≥260 (esophagus)^a^	10-20
			CP	20-40			

Values are presented as mean ± SD. ^a^Anterior or posterior refers to the anterior left atrial wall and posterior left atrial wall, respectively. PVI: pulmonary vein isolation; AI: ablation index; LSI: lesion size index; FTI: force-time integral; HPSD: high-power short-duration; LPLD: low-power long-duration; HPAI: high-power ablation index; CLOSE: CLOSE protocol; HP: high-power; LP: low-power; VHPSD: very-high-power short-duration; MPLD: moderate-power moderate-duration; STSF: Smart Touch Surround Flow (catheter).

## Data Availability

The datasets used and/or analyzed during the current study are available from the corresponding author on reasonable request.

## Supplementary Materials

Supplementary Materials Supplementary materials including searching strategies, figures, and tables can be found in supplementary file_1.
