# Supplementary material for: Comparison between High-Power Short-Duration and Conventional Ablation Strategy in Atrial Fibrillation: An Updated Meta-Analysis
Source: Cardiovasc Ther. 2022 Jul 29;2022:1065077. doi: 10.1155/2022/1065077 (PMC9355769; doi:10.1155/2022/1065077)
Supplement: Supplementary Materials — including searching strategies, figures, and tables can be found in supplementary file_1. [file 1065077.f1.docx]

# Searching strategies

### PubMed

Supplementary Table S1 Searching strategy in PubMed

| Step | Query |
| --- | --- |
| #1 | "Atrial Fibrillation"[Mesh] |
| #2 | ((((((((((((((((((((((((Atrial Fibrillations[Title/Abstract]) OR (Fibrillation, Atrial[Title/Abstract])) OR (Fibrillations, Atrial[Title/Abstract])) OR (Auricular Fibrillation[Title/Abstract])) OR (Auricular Fibrillations[Title/Abstract])) OR (Fibrillation, Auricular[Title/Abstract])) OR (Fibrillations, Auricular[Title/Abstract])) OR (Persistent Atrial Fibrillation[Title/Abstract])) OR (Atrial Fibrillation, Persistent[Title/Abstract])) OR (Atrial Fibrillations, Persistent[Title/Abstract])) OR (Fibrillation, Persistent Atrial[Title/Abstract])) OR (Fibrillations, Persistent Atrial[Title/Abstract])) OR (Persistent Atrial Fibrillations[Title/Abstract])) OR (Familial Atrial Fibrillation[Title/Abstract])) OR (Atrial Fibrillation, Familial[Title/Abstract])) OR (Atrial Fibrillations, Familial[Title/Abstract])) OR (Familial Atrial Fibrillations[Title/Abstract])) OR (Fibrillation, Familial Atrial[Title/Abstract])) OR (Fibrillations, Familial Atrial[Title/Abstract])) OR (Paroxysmal Atrial Fibrillation[Title/Abstract])) OR (Atrial Fibrillation, Paroxysmal[Title/Abstract])) OR (Atrial Fibrillations, Paroxysmal[Title/Abstract])) OR (Fibrillation, Paroxysmal Atrial[Title/Abstract])) OR (Fibrillations, Paroxysmal Atrial[Title/Abstract])) OR (Paroxysmal Atrial Fibrillations[Title/Abstract]) |
| #3 | #1 OR #2 |
| #4 | "Radiofrequency Ablation"[Mesh] |
| #5 | ((((Ablation, Radiofrequency[Title/Abstract]) OR (Radio Frequency Ablation[Title/Abstract])) OR (Ablation, Radio Frequency[Title/Abstract])) OR (Radio-Frequency Ablation[Title/Abstract])) OR (Ablation, Radio-Frequency[Title/Abstract]) |
| #6 | "Catheter Ablation"[Mesh] |
| #7 | (((((((((((((((((((((Ablation, Catheter[Title/Abstract]) OR (Catheter Ablation, Transvenous[Title/Abstract])) OR (Transvenous Catheter Ablation[Title/Abstract])) OR (Ablation, Transvenous Catheter[Title/Abstract])) OR (Catheter Ablation, Electric[Title/Abstract])) OR (Electrical Catheter Ablation[Title/Abstract])) OR (Catheter Ablation, Electrical[Title/Abstract])) OR (Ablation, Electrical Catheter[Title/Abstract])) OR (Electric Catheter Ablation[Title/Abstract])) OR (Ablation, Electric Catheter[Title/Abstract])) OR (Ablation, Transvenous Electric[Title/Abstract])) OR (Electric Ablation, Transvenous[Title/Abstract])) OR (Transvenous Electric Ablation[Title/Abstract])) OR (Ablation, Transvenous Electrical[Title/Abstract])) OR (Electrical Ablation, Transvenous[Title/Abstract])) OR (Transvenous Electrical Ablation[Title/Abstract])) OR (Catheter Ablation, Radiofrequency[Title/Abstract])) OR (Radiofrequency Catheter Ablation[Title/Abstract])) OR (Ablation, Radiofrequency Catheter[Title/Abstract])) OR (Catheter Ablation, Percutaneous[Title/Abstract])) OR (Percutaneous Catheter Ablation[Title/Abstract])) OR (Ablation, Percutaneous Catheter[Title/Abstract]) |
| #8 | (Pulmonary vein isolation[Title/Abstract]) OR (PVI[Title/Abstract]) |
| #9 | #4 OR #5 OR #6 #7 OR #8 |
| #10 | (((((high power[Title/Abstract]) OR (higher power[Title/Abstract])) OR (high-power[Title/Abstract])) OR (high power short duration[Title/Abstract])) OR (high-power short-duration[Title/Abstract])) OR (power[Title/Abstract]) |
| #11 | #3 AND #9 AND #10 |

### Embase

Supplementary Table S2 Searching strategy and results in Embase

| Step | Query | Results |
| --- | --- | --- |
| #1 | 'atrial fibrillation'/exp | 168,565 |
| #2 | 'atrial fibrillations':ab,ti OR 'fibrillation, atrial':ab,ti OR 'fibrillations, atrial':ab,ti OR 'auricular fibrillation':ab,ti OR 'auricular fibrillations':ab,ti OR 'fibrillation, auricular':ab,ti OR 'fibrillations, auricular':ab,ti OR 'persistent atrial fibrillation':ab,ti OR 'atrial fibrillation, persistent':ab,ti OR 'atrial fibrillations, persistent':ab,ti OR 'fibrillations, persistent atrial':ab,ti OR 'fibrillation, persistent atrial':ab,ti OR 'persistent atrial fibrillations':ab,ti OR 'familial atrial fibrillation':ab,ti OR 'atrial fibrillation, familial':ab,ti OR 'atrial fibrillations, familial':ab,ti OR 'familial atrial fibrillations':ab,ti OR 'fibrillation, familial atrial':ab,ti OR 'fibrillations, familial atrial':ab,ti OR 'paroxysmal atrial fibrillation':ab,ti OR 'atrial fibrillation, paroxysmal':ab,ti OR 'atrial fibrillations, paroxysmal':ab,ti OR 'fibrillation, paroxysmal atrial':ab,ti OR 'fibrillations, paroxysmal atrial':ab,ti OR 'paroxysmal atrial fibrillations':ab,ti | 13,563 |
| #3 | #1 OR #2 | 169,180 |
| #4 | 'radiofrequency ablation'/exp | 37,214 |
| #5 | 'ablation, radiofrequency':ab,ti OR 'radio frequency ablation':ab,ti OR 'ablation, radio frequency':ab,ti OR 'radio-frequency ablation':ab,ti OR 'ablation, radio-frequency':ab,ti | 1,630 |
| #6 | #4 OR #5 | 37,754 |
| #7 | 'catheter ablation'/exp | 38,850 |
| #8 | 'ablation, catheter':ab,ti OR 'catheter ablation, transvenous':ab,ti OR 'transvenous catheter ablation':ab,ti OR 'ablation, transvenous catheter':ab,ti OR 'catheter ablation, electric':ab,ti OR 'electrical catheter ablation':ab,ti OR 'catheter ablation, electrical':ab,ti OR 'ablation, electrical catheter':ab,ti OR 'electric catheter ablation':ab,ti OR 'ablation, electric catheter':ab,ti OR 'ablation, transvenous electric':ab,ti OR 'electric ablation, transvenous':ab,ti OR 'transvenous electric ablation':ab,ti OR 'ablation, transvenous electrical':ab,ti OR 'electrical ablation, transvenous':ab,ti OR 'transvenous electrical ablation':ab,ti OR 'catheter ablation, radiofrequency':ab,ti OR 'radiofrequency catheter ablation':ab,ti OR 'ablation, radiofrequency catheter':ab,ti OR 'catheter ablation, percutaneous':ab,ti OR 'percutaneous catheter ablation':ab,ti OR 'ablation, percutaneous catheter':ab,ti | 7,780 |
| #9 | #7 OR #8 | 10,547 |
| #10 | 'pulmonary vein isolation':ab,ti OR 'pvi':ab,ti | 11,417 |
| #11 | #6 OR #9 OR #10 | 76,685 |
| #12 | 'high power':ab,ti OR 'higher power':ab,ti OR 'high-power':ab,ti OR 'high power short duration':ab,ti OR 'high-power short-duration':ab,ti OR 'power':ab,ti | 368,749 |
| #13 | #3 AND #11 AND #12 | 785 |

### Cochrane

Supplementary Table S3 Searching strategy and results in Cochrane Library

| Step | Query | Results |
| --- | --- | --- |
| #1 | Atrial fibrillation | 13,554 |
| #2 | (Atrial Fibrillations):ab,ti,kw OR (Fibrillation, Atrial):ab,ti,kw OR (Fibrillations, Atrial):ab,ti,kw OR (Auricular Fibrillation):ab,ti,kw OR (Auricular Fibrillations):ab,ti,kw OR (Fibrillation, Auricular):ab,ti,kw OR (Fibrillations, Auricular):ab,ti,kw OR (Persistent Atrial Fibrillation):ab,ti,kw OR (Atrial Fibrillation, Persistent):ab,ti,kw OR (Atrial Fibrillations, Persistent):ab,ti,kw OR (Fibrillation, Persistent Atrial):ab,ti,kw OR (Fibrillations, Persistent Atrial):ab,ti,kw OR (Persistent Atrial Fibrillations):ab,ti,kw OR (Familial Atrial Fibrillation):ab,ti,kw OR (Atrial Fibrillation, Familial):ab,ti,kw OR (Atrial Fibrillations, Familial):ab,ti,kw OR (Familial Atrial Fibrillations):ab,ti,kw OR (Fibrillation, Familial Atrial):ab,ti,kw OR (Fibrillations, Familial Atrial):ab,ti,kw OR (Paroxysmal Atrial Fibrillation):ab,ti,kw OR (Atrial Fibrillation, Paroxysmal):ab,ti,kw OR (Atrial Fibrillations, Paroxysmal):ab,ti,kw OR (Fibrillation, Paroxysmal Atrial):ab,ti,kw OR (Fibrillations, Paroxysmal Atrial):ab,ti,kw OR (Paroxysmal Atrial Fibrillations):ab,ti,kw | 13,311 |
| #3 | #1 OR #2 | 13,560 |
| #4 | Radiofrequency Ablation | 3,242 |
| #5 | (Ablation, Radiofrequency):ab,ti,kw OR (Radio Frequency Ablation):ab,ti,kw OR (Ablation, Radio Frequency):ab,ti,kw OR (Radio-Frequency Ablation):ab,ti,kw OR (Ablation, Radio-Frequency):ab,ti,kw | 3,171 |
| #6 | #4 OR #5 | 3,289 |
| #7 | Catheter Ablation | 3,401 |
| #8 | (Ablation, Catheter):ab,ti,kw OR (Catheter Ablation, Transvenous):ab,ti,kw OR (Transvenous Catheter Ablation):ab,ti,kw OR (Ablation, Transvenous Catheter):ab,ti,kw OR (Catheter Ablation, Electric):ab,ti,kw OR (Electrical Catheter Ablation):ab,ti,kw OR (Catheter Ablation, Electrical):ab,ti,kw OR (Ablation, Electrical Catheter):ab,ti,kw OR (Electric Catheter Ablation):ab,ti,kw OR (Ablation, Electric Catheter):ab,ti,kw OR (Ablation, Transvenous Electric):ab,ti,kw OR (Electric Ablation, Transvenous):ab,ti,kw OR (Transvenous Electric Ablation):ab,ti,kw OR (Ablation, Transvenous Electrical):ab,ti,kw OR (Electrical Ablation, Transvenous):ab,ti,kw OR (Transvenous Electrical Ablation):ab,ti,kw OR (Catheter Ablation, Radiofrequency):ab,ti,kw OR (Radiofrequency Catheter Ablation):ab,ti,kw OR (Ablation, Radiofrequency Catheter):ab,ti,kw OR (Catheter Ablation, Percutaneous):ab,ti,kw OR (Percutaneous Catheter Ablation):ab,ti,kw OR (Ablation, Percutaneous Catheter):ab,ti,kw | 3,323 |
| #9 | #7 OR #8 | 3,402 |
| #10 | (Pulmonary vein isolation):ab,ti,kw OR (PVI):ab,ti,kw | 1,632 |
| #11 | #6 OR #9 OR #10 | 5,677 |
| #12 | (high power):ab,ti,kw OR (higher power):ab,ti,kw OR (high-power):ab,ti,kw OR (high power short duration):ab,ti,kw OR (high-power short-duration):ab,ti,kw OR (power):ab,ti,kw | 35,787 |
| #13 | #3 AND #11 AND #12 | 109 |

Supplementary Table S4 Endpoints and the application of antiarrhythmic drug of included studies

| NO. | Study | Endpoints | Antiarrhythmic drug during follow-up |
| --- | --- | --- | --- |
| 1 | Nilsson. 2006 | Complete PV isolation, RF time, total amount of energy, RF applications, audible pop, ablation procedure time (the time between the first and last RF application), fluoroscopic time, radiation dose, patients remained stable SR (sinus rhythm) during follow-up | About half of patients required additional antiarrhythmic drug |
| 2 | Yamada, 2006 | Successful SOCA (segmental ostial catheter ablation), electrical connections, total procedure time, fluoroscopy time, RF energy needed to complete the SOCA, recurrence of PAF at 6 months of follow-up | No antiarrhythmic drugs were administered |
| 3 | Dhillon, 2019 | Procedure duration (time taken from commencing lignocaine injection at the start to removal of the femoral sheaths at the end), fluoroscopy time, radiofrequency ablation time (the time for which RF energy was applied) to achieve PVI, acute PV reconnection, freedom from atrial arrhythmia at 12 months (freedom from AF or atrial tachyarrhythmias lasting greater than 30 seconds after a 3‐month blanking period as per current guidelines), first‐pass isolation (PVI occurring with the completion of the circumferential PVI line), proportion of patients requiring ablation on the intervenous ridge between the PVs to achieve PVI | Proportion of free from recurrent atrial arrhythmia and off antiarrhythmic drugs was collected. |
| 4 | Berte, 2019 | Radiofrequency (RF) time ( RF delivered and not as the time between the first and last ablation lesion formation), fluoroscopy time ( entire use of fluoroscopy), procedure time (the time from the start of the femoral anesthesia to the withdrawal of all sheaths), first pass isolation (both ipsilateral veins were isolated after encircling, and no additional RF lesions were needed), recurrence (any AF, atrial tachycardia or flutter [AT or AFL] >30 seconds during follow-up), complications (vascular and valvular damage, charring on the catheter tip, audible steam pops, perforation with tamponade or atrio‐esophageal fistulae, death, transient ischemic attack, and cerebrovascular attack occurred within 7 days and related to the procedure when they occurred within the first month after the ablation procedure) | All class 1c and class III antiarrhythmic drugs (AADs) remained stopped after ablation. |
| 5 | Okamatsu, 2019 | Time to complete CPVI, first‐pass isolation (creation of a bidirectional block between the LA and PV after the initial circumferential ablation), fluoroscopy time, esophageal temperature parameters, VisiTag data of RF point applied to circular, RF application time, average CF, FTI, and AI for PV antrum and intervenous carina, percentage of VisiTag points achieving target AI, AF recurrence (any atrial tachyarrhythmias lasting for more than 30 seconds occurring after a 3‐month blanking period), complete CPVI, time to complete the circular RF application, RF power application, LA‐PV reconnection after CPVI, time to complete CPVI for both sides, procedure time, esophageal temperature, ablation procedure‐related complications | N/A |
| 6 | Pambrun, 2019 | Recurrence (any documented episode of AF or atrial tachycardia lasting >30 s) at 12 months, major complications related to the lesions (tamponade, stroke, esophageal fistula), groin hematoma, procedure time, ablation time, energy delivery, first-pass PVI(exit block obtained after initial anatomic encirclement; exit block was defined as the failure to capture the left atrium while pacing with the ablation catheter within the antrum, carina included), PV reconnections, fluoroscopy time, ablation time per point, total energy delivery | Antiarrhythmic drugs were systematically discontinued. |
| 7 | Vassallo, 2019 | Mean time for the left atrium, mean total procedure time, mean RF time, time of radioscopy, time in LA, total procedure time, radiofrequency time, fluoroscopy time, elevation of esophageal temp, capacity to isolate PVI before completing the total circumference of these vessels, 6‐month and 12-month follow‐up recurrences | Antiarrhythmics were maintained for 90 days. |
| 8 | Castrejón-Castrejón, 2019 | Circumferential radiofrequency delivery completed (ipsilateral veins were surrounded by a line of contiguous target lesions), PV reconnection (recovery of PV conduction following 5 min of the last radiofrequency application needed to achieve PVI, in general evaluated at the end of the procedure), total ablation procedural time, fluoroscopy time, total radiofrequency time, hospitalization stay, isolation of all targeted PVs, Fist-pass PVI ( PVI was demonstrated following complete circumferential radiofrequency delivery), PV reconnection following PVI, radiofrequency applications, radiofrequency delivery time, LSI for lesion, impedance drop, esophageal lesions, other complications (cardiac tamponade, pericardial effusion), audible steam pops | N/A |
| 9 | Ejima, 2019 | Complete CPVI, procedural time, fluoroscopic time, RF time, ablation time per point during the PVI, time‐dependent acute PVRs, ATP‐induced dormant conduction, acute PVR, ATA recurrence (any episode of ATAs lasting >30 seconds after a 2‐month blanking period from the ablation procedure without any AADs), complications (asymptomatic phrenic nerve injury, gastric hypomotility, atrio‐esophageal fistulae, cardiac tamponades, strokes) | AADs were prescribed only if any early recurrences of an ATAs were observed before discharge and were discontinued by 3 months after the procedure. |
| 10 | Kottmaier, 2020 | PV reconnection, vascular complications (hematoma <5 cm, hematoma >5 cm, pseudoaneurysm, arteriovenous fistula), mean RF time, procedural duration, fluoroscopy time, fluoroscopy dose, dormant conduction, overall applied energy, minimal, maximal, and mean applied current per lesion, impedance drop ,complications (pericardial tamponade and no periprocedural thromboembolic complications, atrio-oesophageal fistula), AF recurrences during the 1-year follow-up, atrial tachycardia (AT) recurrence during the 1-year follow-up | Antiarrhythmic drug therapy detail not mentioned, while off-antiarrhythmic and free from AT was reported |
| 11 | Kyriakopoulou, 2019 | Ablation procedure time, fluoroscopy time, radiation dose, dose area product, total PVI RF time, the time to isolate the PVs, number of RF applications, RF time per application, number of dislocations, First‐pass isolation, reconnection rate after adenosine, procedural complication (steam pop, cardiac perforation, stroke, death), ATA recurrence within the first year (any atrial tachyarrhythmia (ATA) > 30 seconds on Holter at 1 year or earlier on the anamnestic indication) | Antiarrhythmic drug therapy was left at the discretion of the treating physician at ATA recurrence. |
| 12 | Leo, 2020 | **Primary endpoints:** Occurrence and number of ETAs (esophageal temperature alert) per patient during radiofrequency ablation on the LA posterior wall.  **Secondary endpoints:** Rate of first-pass PVI (PVI achieved at completion of encirclement around the PVs), rate of acute PV reconnection (reappearance of PV potentials or conduction into the LA during PV pacing during the index procedure, observed in the waiting time soon after successful isolation), total procedural time, total radiofrequency time, and percentage of radiofrequency lesions reaching the target LSI on the LA posterior wall. AF recurrence was defined as the occurrence of symptomatic atrial tachyarrhythmias lasting >30 seconds was defined as AF recurrence | Antiarrhythmic drugs were discontinued after the procedure. |
| 13 | Shin, 2020 | Successful PVI, procedural time, ablation time, fluoroscopy times, the number of ablation lesions, mean CF, AI, cardiac tamponade, clinical ischemic stroke or death, clinical recurrence. | Antiarrhythmic drugs were allowed for the first 3 months but then discontinued if SR was maintained. |
| 14 | Yavin, 2020 | Successful PVI (loss of PV potentials and failure to capture the left atrium during pacing from all bipoles of a multielectrode mapping catheter positioned at the PV ostium), acute PV reconnection (recurrence of PV potentials or ability to capture the left atrium during pacing from the multielectrode mapping catheter positioned at the PV ostium within 20 min after complete PVI), recurrent sustained atrial arrhythmia (sustained AF or atrial tachycardia >30s occurring ≥4 weeks after the ablation procedure RF application), duration, impedance drop, contact force, catheter stability during energy delivery, total RF time, frequency of achieving PVI after initial encirclement (first pass), the transpired ablation time (the time from the first to last ablation application required for PVI ), safety parameters (steam pops, catheter char, pericardial effusion, phrenic nerve paralysis, maximal esophageal temperature, atrioesophageal fistulas, transient ischemic attack, stroke, death) | N/A |
| 15 | Yazaki, 2020 | ATA recurrence during follow-up (recurrent symptoms and/or detection of ATAs using the several aforementioned modalities or data provided by cardiac implantable electrical devices lasting > 30s after a 2-month blanking period from the ablation procedure without any AADs), acute PV reconnection (spontaneous or isoproterenol induced PVR, or adenosine-induced dormant conduction), RF time, RF energy, total isolation time, total procedure time, bilateral isolation length, radiation exposure, per-segment analysis (minimum contact force, minimum force-time integral, minimum ablation index, maximum inter-lesion distance, minimum impedance drop) | If AAD was used for patients after the procedure, it was considered as “ATA recurrence.” |
| 16 | Dikdan, 2021 | **Primary procedural endpoints:** Total ablation time (TAT, the summation of both LPVT and RPVT), left pulmonary vein isolation time (LPVT, the time taken to isolate the left PVs beginning with the first RF lesion and ending with the final RF lesion), right pulmonary vein isolation time (RPVT, the time to isolate the right PVs in the same manner), RF ablation delivery time (RADT, total time that RF ablation was delivered and not the time in between lesions), and the inducibility of arrhythmias after ablation.  **Primary clinical endpoints:** recurrence of AF (≥30 s of asymptomatic or symptomatic AF) in the first 3 months and 12 months after ablation, the probability of AF recurrence over 12 months by Kaplan–Meier survival analysis. | N/A |
| 17 | Wielandts, 2021 | Total procedural time, fluoroscopy time, Air Kerma, PVI procedure time, total radiofrequency application time necessary for PVI, total number of radiofrequency applications for PVI, first pass PVI, adenosine induced reconnection, maximum reached temperature, average CF, impedance drop, complete PV isolation, acute procedural complications, steam pops, esophageal lesion, 6-months recurrence of AF-related tachy-arrhythmias | Antiarrhythmic drug treatment was discontinued at 1-month follow-up. |
| 18 | Francke, 2021 | **Primary efficiency endpoints:** differences in total procedural time, total RF application time, time to PVI  **Primary safety endpoint:** number and characteristics of thermal esophageal lesions (EDELs)  **Secondary efficiency endpoint:** the number of required RF lesions, RF time per lesion, the number of patients with the first-pass PVI, the AF recurrence rate within the first 12 weeks after the ablation procedure  **Secondary safety endpoint:** occurrence of procedural related complications other than thermal EDELs | N/A |
| 19 | Hansom, 2021 | **The primary endpoint:** freedom from AAs ≥ 30 s in duration at 1 year, excluding a 3‐month blanking period immediately post procedure  **Secondary endpoints:** rates of acute reconnection, rates of adenosine‐induced reconnection, safety outcomes (major bleeding, vascular access complications, cerebrovascular accidents, pericardial tamponade, atrioesophageal fistula, and death), and procedure duration. | Typically stopped at 3 months post ablation but was left to physician discretion.  Of note, four patients in the HPSD group and five patients in the LPLD group remained on antiarrhythmic medications without recurrence. |
| 20 | Richard, 2021 | Successful PV isolation, first pass PV isolations, procedure times, LA dwelling times, fluoroscopy times, total number of applications, total ablation time, mean application duration, mean power per application, total delivered energy per lesion, major periprocedural complications (major groin bleeding, cardiogenic shock, pulmonary edema, cardiac tamponade, pericardial effusion, stroke, phrenic nerve palsy or atrioesophageal fistula, steam pops) | Previously ineffective antiarrhythmic drugs or a new antiarrhythmic drug were prescribed and continued for 3 months post ablation. |
| 21 | Lee, 2021 | Acute PV reconnection (the presence of early reconnection after a 20- minute observation of PVI achievement), total procedure time, total ablation time, ablation time for PVI, fluoroscopy time, rate of RP, acute PVR among PV segments,  **The long- term efficacy endpoint:** the recurrence of AT or AF evaluated by documented any AT or AF lasting longer than 30 seconds within 1- year follow-up after the 3-month blanking period after the index procedure.  **Safety outcomes:** procedure-related complications (steam pop, cardiac tamponade, atrial- esophageal fistula, stroke, transient ischemic attacks, and death within 4-weeks and 1year after the index procedure) | N/A |
| 22 | Okamatsu, 2021 | Procedure time, percentage of first-pass PV isolations (creating a bidirectional block between the LA and PV after the initial circumferential ablation), tachyarrhythmia recurrence-free survival curves, the respective 1-year atrial tachyarrhythmia recurrence-free survival rates (AF recurrence was defined as any atrial tachyarrhythmias lasting >30 s that occurred after a 3-month blanking period), procedure-related complications (death, perforation/tamponade, steam pop, atrioesophageal fistula, endoscopic esophageal lesion, PV stenosis, phrenic nerve injury, gastric hypomotility, stroke/thromboembolic events, puncture site hematoma/vascular injury, heart failure, pericarditis, nasal bleeding related to nasal airway device),. Characteristics related to Redo procedures | N/A |

|  | Selection | | | | Comparability | Outcome | | |
| --- | --- | --- | --- | --- | --- | --- | --- | --- |
|  | Representative-ness of the exposed cohort | Selection of the non exposed cohort | Ascertainment of exposure | Demonstration that outcome of interest was not present at start of study | Comparability of cohorts on the basis of the design or analysis | Assessment  of  outcome | Was follow-up long enough for outcomes to occur | Adequacy of follow up of cohorts |
| Nilsson, 2006 | * | * | * | * | * | * | * | * |
| Yamada, 2006 | * | * | * | * | ** | * | - | * |
| Dhillon, 2019 | * | * | * | * | - | * | * | * |
| Berte, 2019 | * | * | * | * | ** | * | - | * |
| Okamatsu, 2019 | * | * | * | * | * | * | - | * |
| Pambrun, 2019 | * | * | * | * | ** | * | * | * |
| Vassallo, 2019 | * | * | * | * | ** | * | * | * |
| Castrejón-Castrejón, 2020 | * | * | * | * | - | * | - | * |
| Ejima, 2020 | * | * | * | * | * | * | * | * |
| Kottmaier, 2020 | * | * | * | * | ** | * | * | * |
| Kyriakopoulou, 2020 | * | * | * | * | * | * | * | * |
| Leo, 2020 | * | * | * | * | * | * | * | * |
| Shin, 2020 | * | * | * | * | - | * | * | * |
| Yavin, 2020 | * | * | * | * | - | * | * | * |
| Yazaki, 2020 | * | * | * | * | - | * | * | * |
| Dikdan, 2021 | * | * | * | * | ** | * | * | * |
| Wielandts, 2021 | * | * | * | * | ** | * | - | * |
| Francke, 2021 | * | * | * | * | ** | * | - | - |
| Hansom, 2021 | * | * | * | * | * | * | * | * |
| Richard, 2021 | * | * | * | * | * | * | - | * |
| Lee, 2021 | * | * | * | * | * | * | * | * |
| Okamatsu, 2021 | * | * | * | * | ** | * | * | - |

Supplementary Table S5 Quality assessment of included studies with Newcastle-Ottawa scale (NOS)

Supplementary Table S6 Meta regression for each endpoint by study design

| Endpoint | Dependent variable | Independent variable | exp(b) | Std. Err. | t | P>t | [95% Conf. | Interval] |
| --- | --- | --- | --- | --- | --- | --- | --- | --- |
| First-pass PVI | logrr | Study Design | 1.016942 | 0.079268 | 0.22 | 0.833 | 0.8566201 | 1.207269 |
|  |  | _cons | 1.086341 | 0.105456 | 0.85 | 0.412 | 0.8773536 | 1.34511 |
| Acute PV reconnection | logrr | Study Design | 0.988519 | 0.492244 | -0.02 | 0.982 | 0.3340343 | 2.925357 |
|  |  | _cons | 0.565142 | 0.331937 | -0.97 | 0.35 | 0.1571731 | 2.032061 |
| Free from AF (6 months) | logrr | Study Design | 0.81393 | 0.08102 | -2.07 | 0.175 | 0.5303745 | 1.249083 |
|  |  | _cons | 1.475169 | 0.240975 | 2.38 | 0.14 | 0.7304588 | 2.979119 |
| Free from AT (6 months) | logrr | Study Design | 0.882166 | 0.101479 | -1.09 | 0.355 | 0.6117297 | 1.272159 |
|  |  | _cons | 1.255784 | 0.201151 | 1.42 | 0.25 | 0.7542708 | 2.090752 |
| Free from AF (12 months) | logrr | Study Design | 0.978477 | 0.111873 | -0.19 | 0.855 | 0.7396902 | 1.294349 |
|  |  | _cons | 1.201312 | 0.176989 | 1.24 | 0.26 | 0.8377055 | 1.722743 |
| Free from AT (12 months) | logrr | Study Design | 1.027347 | 0.096807 | 0.29 | 0.78 | 0.8366666 | 1.261484 |
|  |  | _cons | 1.076365 | 0.115019 | 0.69 | 0.504 | 0.8527959 | 1.358545 |
| Esophageal injury | logrr | Study Design | 1.045852 | 1.655179 | 0.03 | 0.982 | 1.930E-09 | 5.660E+08 |
|  |  | _cons | 0.914238 | 1.811959 | -0.05 | 0.971 | 1.060E-11 | 7.900E+10 |
| Major complications | logrr | Study Design | 1.931745 | 3.308351 | 0.38 | 0.716 | 0.0236589 | 157.7264 |
|  |  | _cons | 0.397908 | 0.778095 | -0.47 | 0.657 | 0.0026105 | 60.65165 |
| Total procedure time | _ES | Study Design | 0.001633 | 0.027255 | -0.38 | 0.716 | 3.77E-22 | 7.07E+15 |
|  |  | _cons | 2.15E-12 | 4.39E-11 | -1.32 | 0.245 | 3.76E-35 | 1.23E+11 |
| PVI time | _ES | Study Design | 0.083914 | 0.700836 | -0.3 | 0.774 | 3.63E-10 | 1.94E+07 |
|  |  | _cons | 4.85E-09 | 4.48E-08 | -2.07 | 0.072 | 2.71E-18 | 8.686904 |
| PVI RF time | _ES | Study Design | 66.49867 | 204.6741 | 1.36 | 0.198 | 0.0813564 | 54354.37 |
|  |  | _cons | 2.41E-09 | 9.16E-09 | -5.23 | 0 | 6.17E-13 | 9.44E-06 |

#
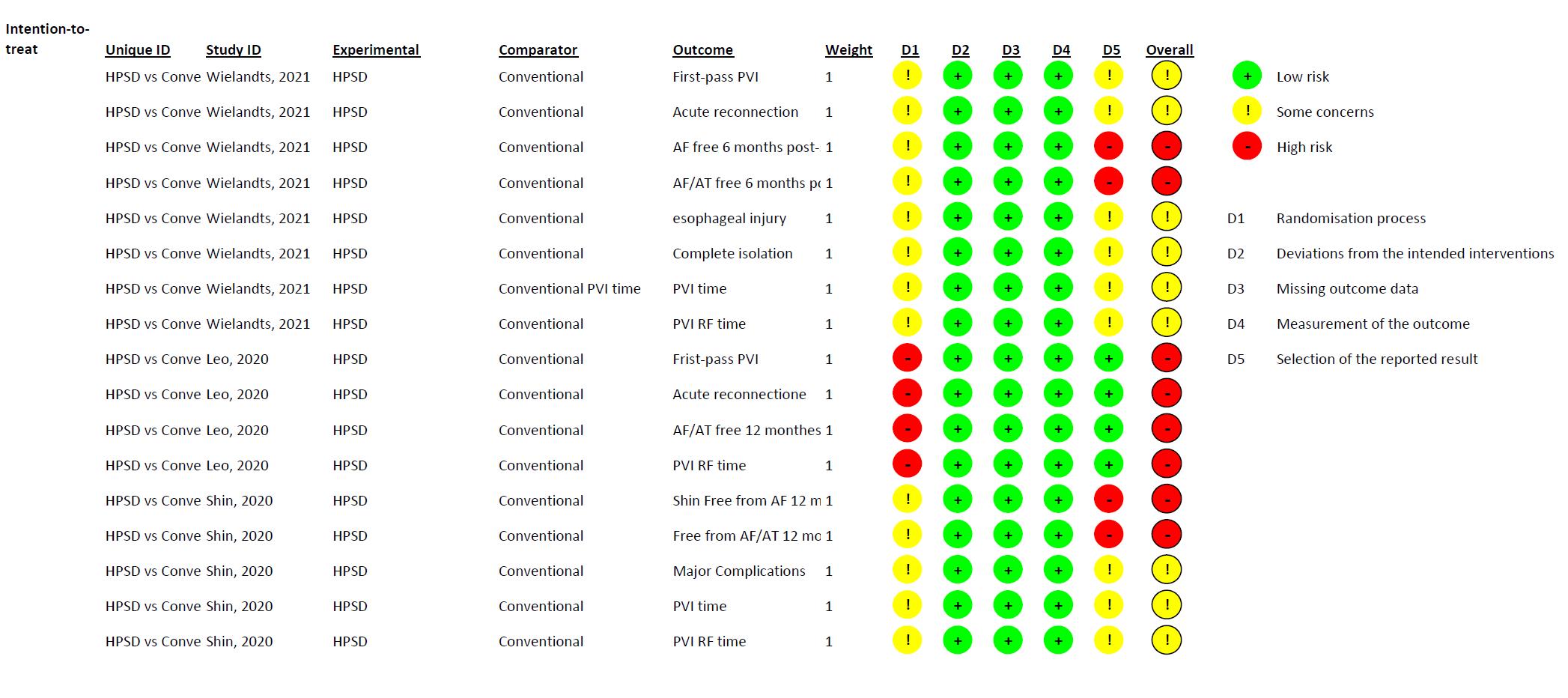
Supplementary figures

Supplementary Figure S1 Traffic light plot demonstrating the risk of bias based on RoB2 (Cochrane risk-of-bias tool for randomized trials) of included randomized control trials. Conven: conventional.

Supplementary Figure S2 Forest plot of pooled effect demonstrating (a) free from atrial fibrillation AF and (b) free from atrial tachycardia (AT) rate 6 months after surgery of high-power short-duration (HPSD) and conventional ablation settings. 95% CI, 95% confidence interval.
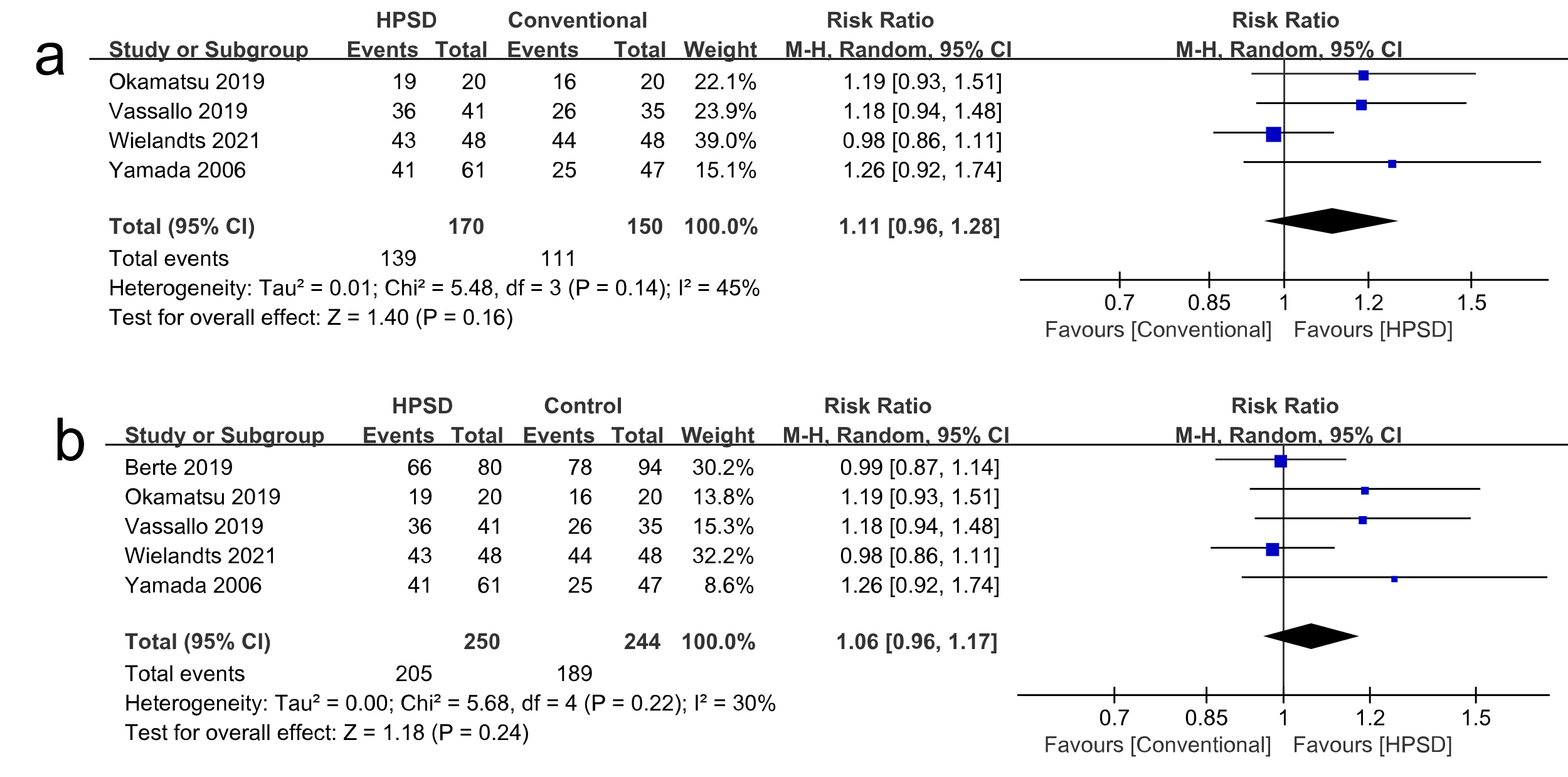


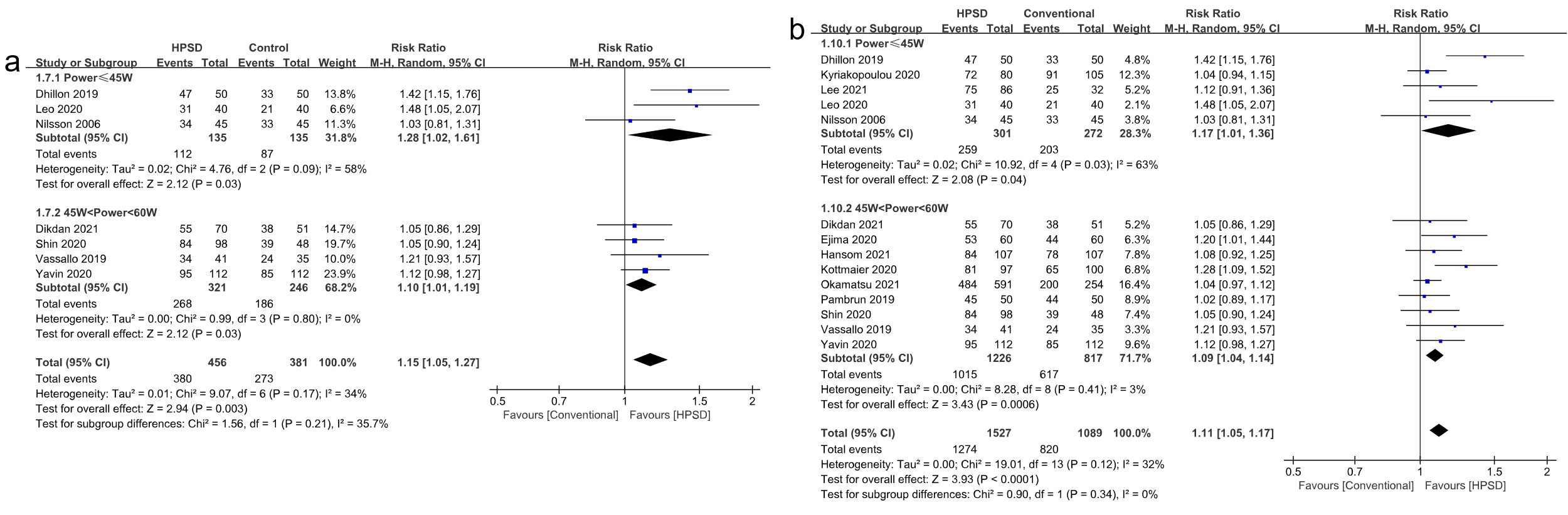


Supplementary Figure S3 Forest plot of pooled effect demonstrating (a) free from atrial fibrillation AF and (b) free from atrial tachycardia (AT) rate 12 months after surgery of high-power short-duration (HPSD) and conventional ablation settings. Subgroup analyses were performed according to ablation power setting. 95% CI, 95% confidence interval.


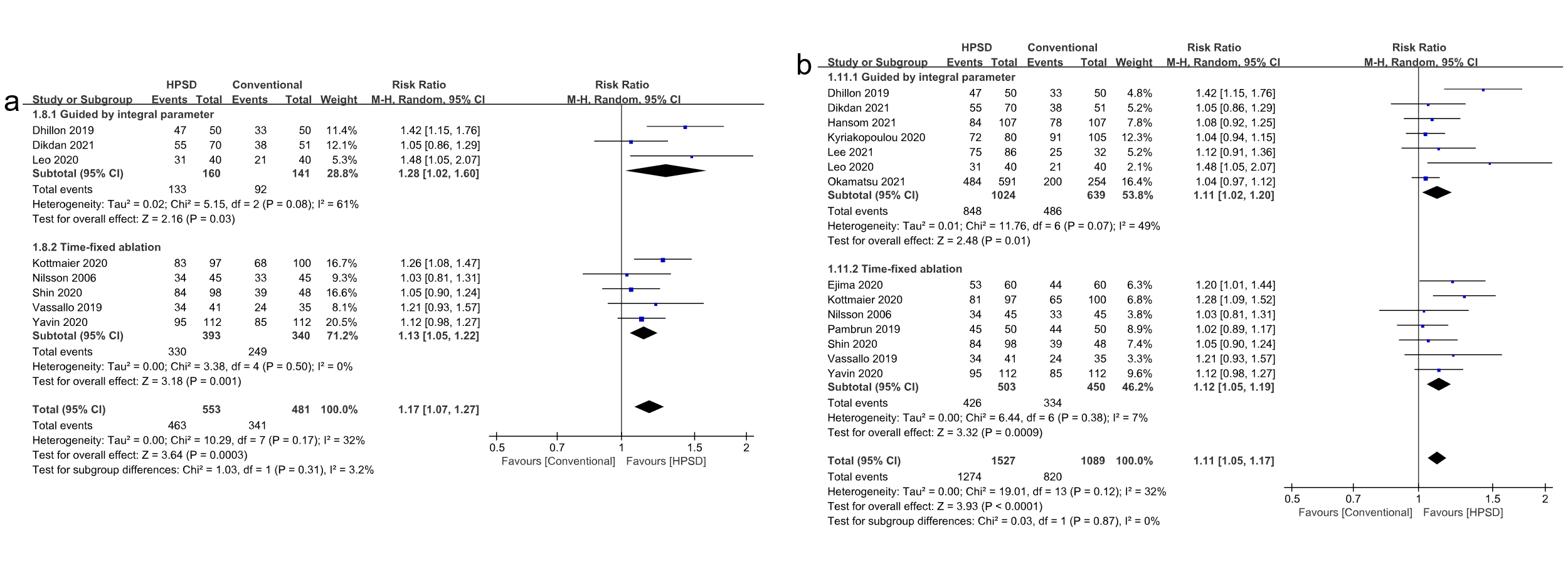


Supplementary Figure S4 Forest plot of pooled effect demonstrating (a) free from atrial fibrillation AF and (b) free from atrial tachycardia (AT) rate 12 months after surgery of high-power short-duration (HPSD) and conventional ablation settings. Subgroup analyses were performed according to ablation guided by integral indexes or fixed time. 95% CI, 95% confidence interval.


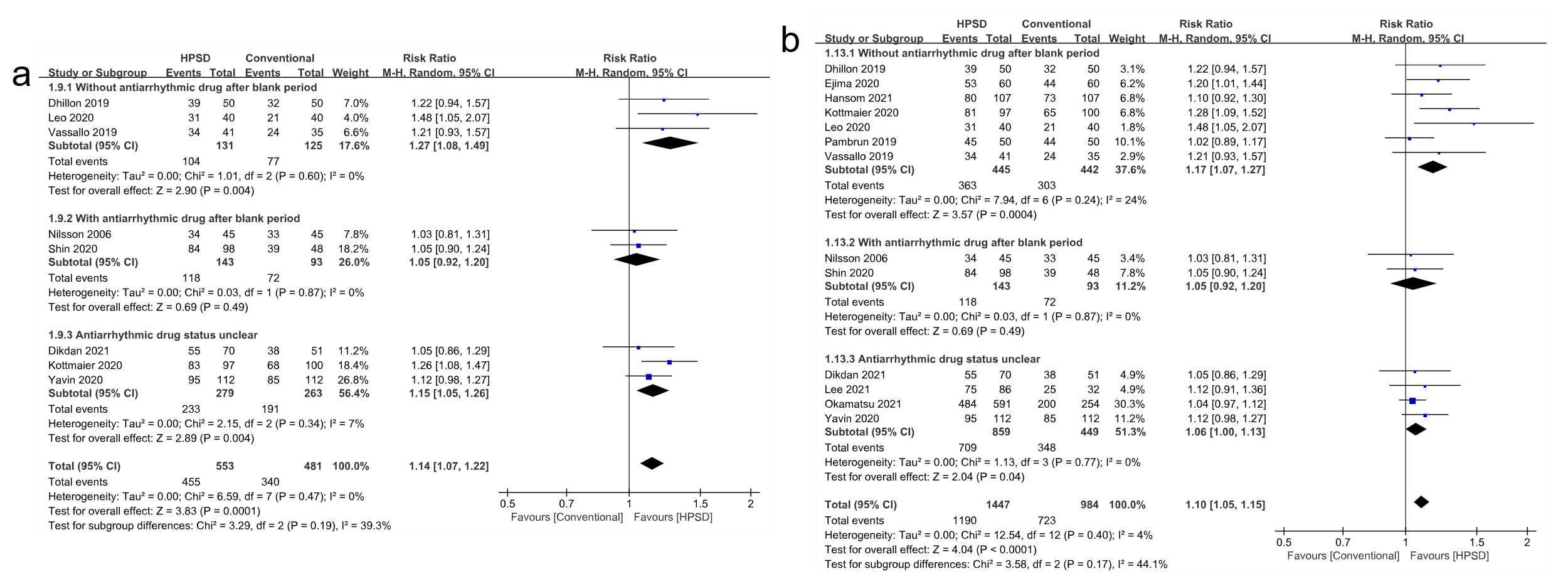


Supplementary Figure S5 Forest plot of pooled effect demonstrating (a) free from atrial fibrillation AF and (b) free from atrial tachycardia (AT) rate 12 months after surgery of high-power short-duration (HPSD) and conventional ablation settings. Subgroup analyses were performed according to the application status of antiarrhythmic drug during follow-up and after three-month blank period. 95% CI, 95% confidence interval.


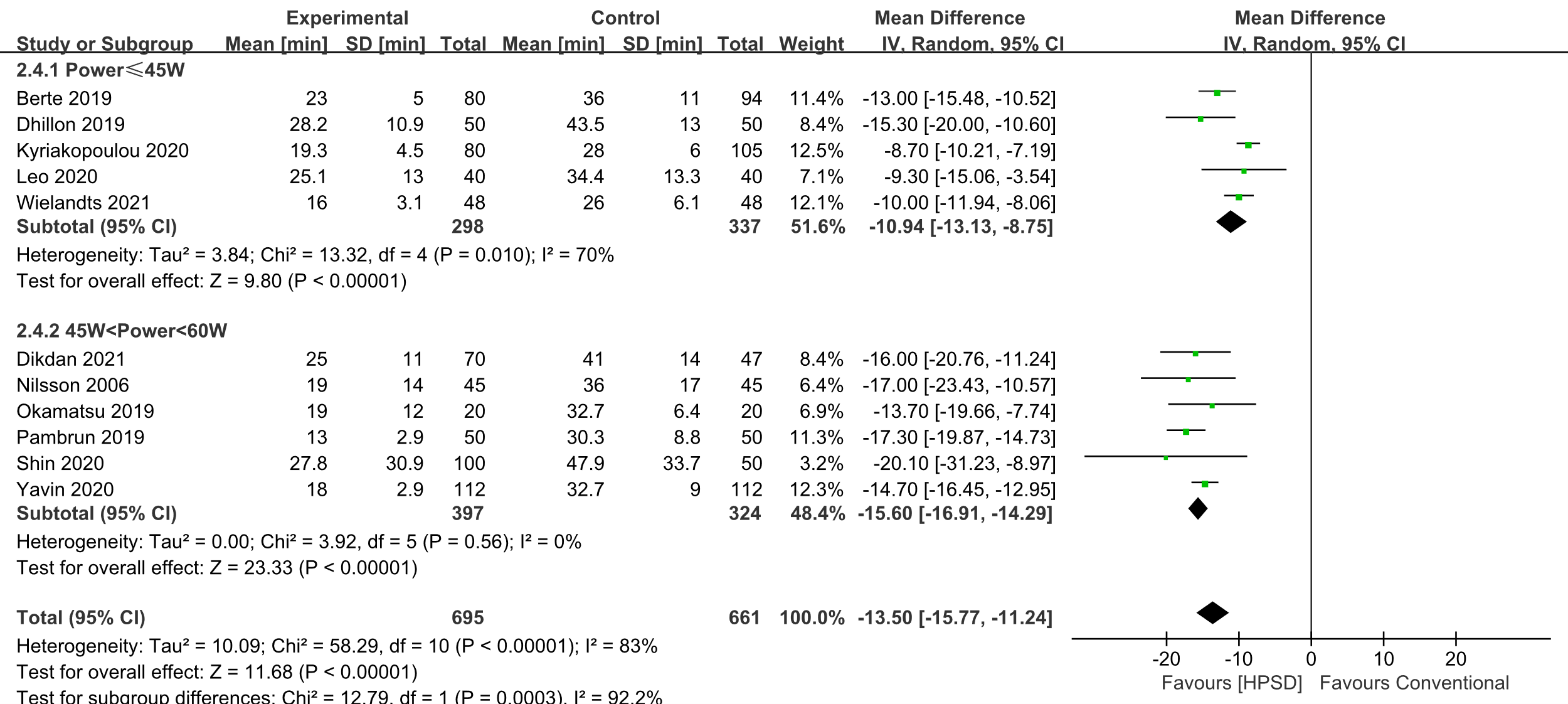


Supplementary Figure S6 Forest plot of pooled effect demonstrating pulmonary vein isolation (PVI) time of high-power short-duration (HPSD) and conventional ablation settings. Subgroup analyses were performed according to ablation power setting. 95% CI, 95% confidence interval.


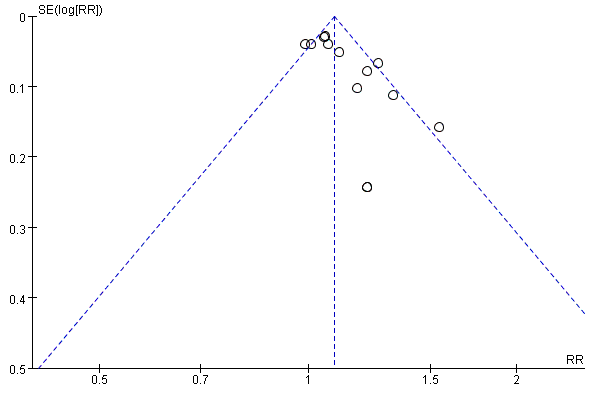


Supplementary Figure S7 Funnel plot of pooled effect demonstrating the first-pass pulmonary vein isolation rate of high-power short-duration (HPSD) and conventional ablation settings.


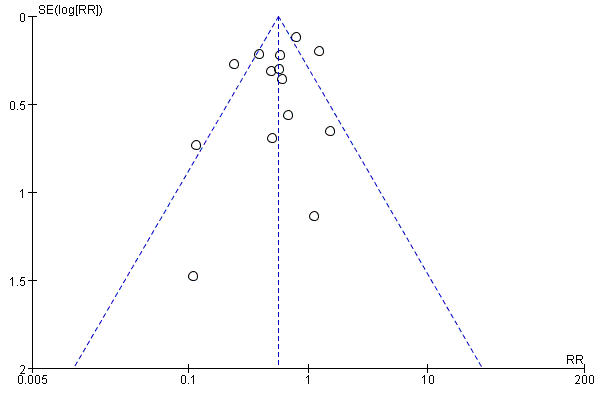


Supplementary Figure S8 Funnel plot of pooled effect demonstrating acute pulmonary vein reconnection of high-power short-duration (HPSD) and conventional ablation settings.


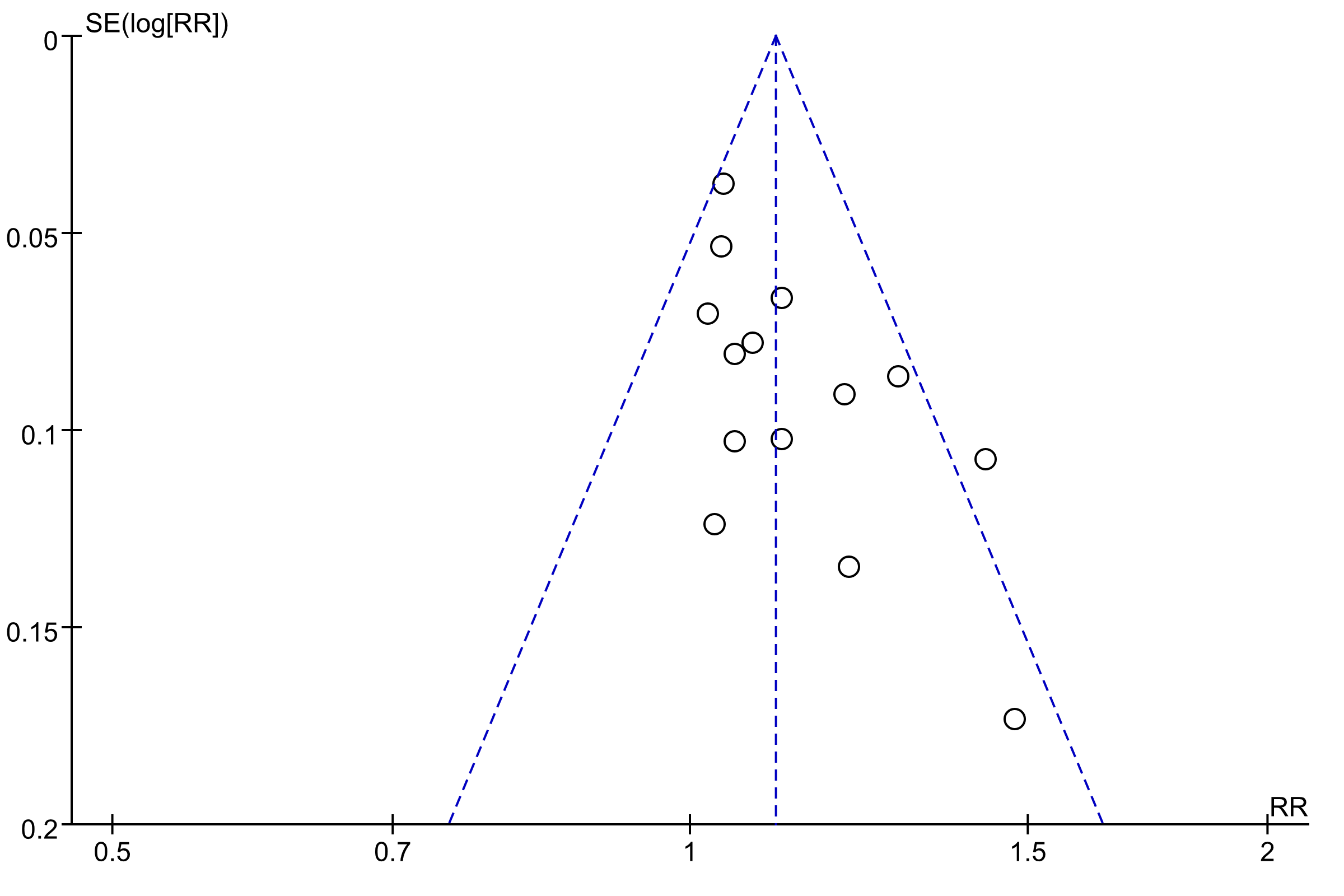


Supplementary Figure S9 Funnel plot of pooled effect demonstrating free from AT 12-month after surgery in high-power short-duration (HPSD) and conventional ablation settings.


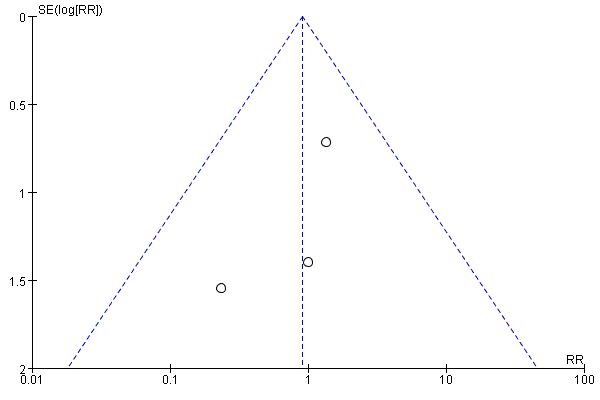


Supplementary Figure S10 Funnel plot of pooled effect demonstrating esophageal injury in high-power short-duration (HPSD) and conventional ablation settings. 95% CI, 95% confidence interval.


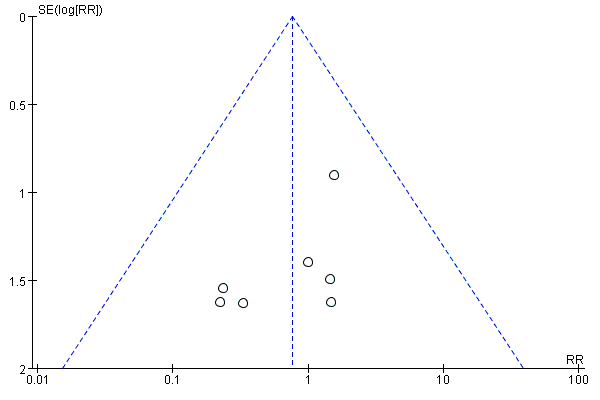


Supplementary Figure S11 Funnel plot of pooled effect demonstrating major complications in high-power short-duration (HPSD) and conventional ablation settings. 95% CI, 95% confidence interval.


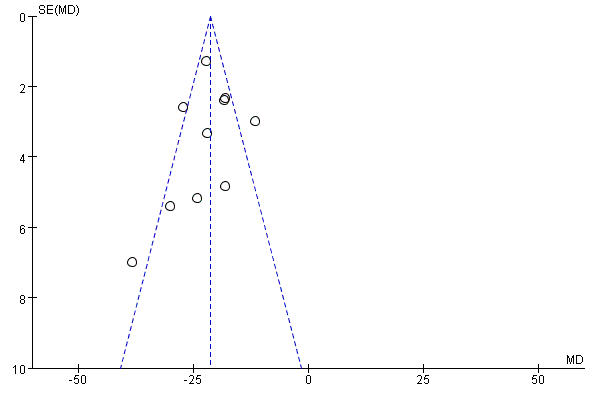


Supplementary Figure S12 Funnel plot of pooled effect demonstrating pulmonary vein isolation (PVI) time in high-power short-duration (HPSD) and conventional groups. IV, inverse variance; 95% CI, 95% confidence interval.


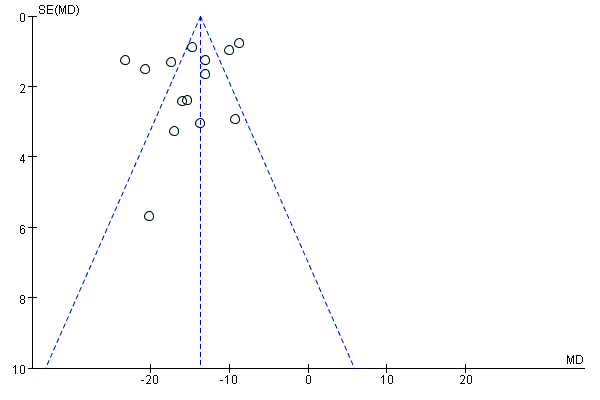


Supplementary Figure S13 Funnel plot of pooled effect demonstrating radiofrequency ablation applied time during pulmonary vein isolation in high-power short-duration (HPSD) and conventional groups. IV, inverse variance; 95% CI, 95% confidence interval.
